# Disorder to order: how halide mixing in MAPbI_3−*x*_Br_*x*_ perovskites restricts MA dynamics†

**DOI:** 10.1039/d2ta09069d

**Published:** 2023-02-01

**Authors:** Kostas Fykouras, Jonathan Lahnsteiner, Nico Leupold, Paul Tinnemans, Ralf Moos, Fabian Panzer, Gilles A. de Wijs, Menno Bokdam, Helen Grüninger, Arno P. M. Kentgens

**Affiliations:** a Faculty of Science and Technology and MESA+ Institute for Nanotechnology, University of Twente P.O. Box 217 7500 AE Enschede Netherlands m.bokdam@utwente.nl; b Department of Functional Materials, University of Bayreuth Universitätsstraße 30 95447 Bayreuth Germany; c Radboud University, Institute for Molecules and Materials Heyendaalseweg 135 6525 AJ Nijmegen Netherlands; d Soft Matter Optoelectronics, University of Bayreuth Universitätsstraße 30 95447 Bayreuth Germany; e Inorganic Chemistry III and Northern Bavarian NMR Centre, University of Bayreuth Universitätsstraße 30 95447 Bayreuth Germany helen.grueninger@uni-bayreuth.de

## Abstract

Mixed-halide lead perovskites are of particular interest for the design of tandem solar cells currently reaching record efficiencies. While halide phase segregation upon illumination of mixed perovskites is extensively studied, the effect of halide disorder on A cation dynamics is not well understood, despite its importance for charge carrier diffusion and lifetime. Here, we study the methylammonium (MA) reorientational dynamics in mixed halide MAPbI_3−*x*_Br_*x*_ perovskites by a combined approach of experimental solid-state NMR spectroscopy and molecular dynamics (MD) simulations based on machine-learning force-fields (MLFF). ^207^Pb NMR spectra indicate the halides are randomly distributed over their lattice positions, whereas PXRD measurements show that all mixed MAPbI_3−*x*_Br_*x*_ samples are cubic. The experimental ^14^N spectra and ^1^H double-quantum (DQ) NMR data reveal anisotropic MA reorientations depending on the halide composition and thus associated disorder in the inorganic sublattice. MD calculations allow us to correlate these experimental results to restrictions of MA dynamics due to preferred MA orientations in their local Pb_8_I_12−*n*_Br_*n*_ “cages”. Based on the experimental and simulated results, we develop a phenomenological model that correlates the ^1^H dipolar coupling and thus the MA dynamics with the local composition and reproduces the experimental data over the whole composition range. We show that the dominant interaction between the MA cations and the Pb–X lattice that influences the cation dynamics is the local electrostatic potential being inhomogeneous in mixed halide systems. As such, we generate a fundamental understanding of the predominant interaction between the MA cations and the inorganic sublattice, as well as MA dynamics in asymmetric halide coordinations.

## Abstract 2

This work reveals the correlation between anisotropy in MA^+^ reorientation dynamics and the nearest-neighbour halide layout, which is associated with the composition in mixed lead halide MAPbI_3−*x*_Br_*x*_ perovskites.

## Graphical abstract

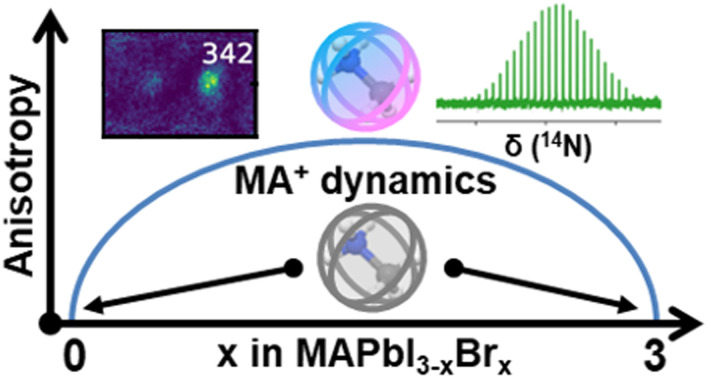

## 1. Introduction

Halide perovskites with their ABX_3_ crystal structure (A: cation, *e.g.*, methylammonium (MA^+^); B: Metal, *e.g.*, Pb^2+^; X: Halide, *e.g.*, I^−^, Br^−^) are currently considered one of the most promising semiconductor materials for the next generation of solar cells. This is due to their excellent optoelectronic properties, such as a high absorption coefficient, along with decent charge carrier diffusion lengths and a high defect tolerance.^1,2^ Moreover, it is possible to change the perovskite's band gap simply by varying the composition, especially of the halides.^3,4^ This allows for controlled optimization of the band gap for the desired application, *e.g.*, in tandem solar cells, where, by combining silicon and mixed perovskites of the form APbI_3−*x*_Br_*x*_, tandem cell efficiencies exceeding 30% have been realized.^5^

Hybrid halide perovskites represent a class of “soft” materials allowing for a suite of molecular and ionic motions.^6,7^ These are probably fundamental for their high opto-electronic performance,^8–11^ but also result in a complex interplay of structure, composition and motional processes on multiple timescales. Both halide and A cation migration are rather slow – on the millisecond to second timescale – and are mostly relevant for long-term stability.^11–14^ Especially halide migration and phase segregation under solar cell working conditions, *i.e.*, under light irradiation,^15–17^ result in heterogeneities in the crystalline structure and thus in significant changes in charge carrier dynamics,^18^ deterioration of the optoelectronic performance and low temporal stability of the perovskite material and optoelectronic devices made thereof.^19^ Cooperative vibrational motions of the lattice (phonons) and A cation reorientation processes are much faster and occur on the pico-second timescale.^7,8,10,20–22^ Both dynamical processes were linked to long charge-carrier lifetimes,^22^ where the role of A cation reorientations was discussed through phonon coupling,^8^ polaron formation^9,23^ and Rashba splitting.^24^

For mixed ion, *i.e.*, mixed halide and/or mixed cation, perovskite systems both static and dynamic disorder occur in addition. These systems are prone to compositional inhomogeneities ranging from the nm-scale up to domains in the sub-μm range as was recently shown for, *e.g.*, mixed cation MA_1−*x*_FA_*x*_PbI_3_ (refs. 25 and 26). Despite the clear importance of cation dynamics, this has only been investigated in-depth for single-halide MAPbX_3_ systems,^22,27–30^ while relatively little is known for the mixed halide systems and even less for high-performance mixed-cation and mixed-halide Pb-based systems, *e.g.*, MA_1−*y*_FA_*y*_PbI_3−*x*_Br_*x*_.^31–35^ Local structural variations potentially have significant impact on the A cation dynamics. Especially the interaction with the inorganic Pb–X sublattice, and heterogeneities therein will impact organic cation dynamics and thus the opto-electronic properties. Therefore, it is essential to study the nature of the cation–halide interactions and connect them to the locally heterogenous structure in mixed perovskite systems. These interactions can be governed by the electrostatic potential, hydrogen bonding, or van der Waals interactions, or a complex interplay thereof.^36–39^

While a range of theoretical and experimental methods are available to study the cation dynamics, *e.g.*, quasi-elastic neutron scattering,^22,35^ calculations,^40–42^ and dielectric^30^ or vibrational spectroscopy,^32,43^ solid-state NMR spectroscopy can simultaneously probe both the local structure of the inorganic sublattice and the MA dynamics.^44–48^ The local halide distributions are directly accessible *via*^207^Pb NMR spectroscopy, as a change in the octahedral Pb–X coordination(s) results in significant changes of the ^207^Pb chemical shift.^49–52^ As such, it can quantitatively probe the formation of mixed halide phases on the atomic scale, *i.e.*, determine whether the populations of the different Pb–X resonances follow a random (binomial) distribution expected for solid-solutions, or whether compositional fluctuations due to a phase segregation of ions on the macro- and nanoscale occur.^49^ This information is often complemented by XRD techniques to corroborate the “average” (long range) crystal structure of the mixed systems and probe macroscopic ion mixing or phase segregation.^53,54^^14^N and ^2^H NMR spectroscopy are sensitive to dynamics of the organic cations, such as MA^+^ and FA^+^ tumbling, because these motions affect (a) the (characteristic) time average of the anisotropic NMR interactions, *i.e.*, the quadrupolar interaction, and (b) the relaxation behaviour of the quadrupolar nuclei.^27–29,44,55,56^ While the time averaging provides information about the apparent symmetry of the cation sites, the spin relaxation is sensitive to the motion on the timescale of the nuclear Larmor frequency.^44^ A connection of experimental NMR spectroscopic results to structural motifs is often obtained by first-principles calculations.^57–62^ Especially useful are molecular-dynamics (MD) simulations that accurately describe dynamic systems. Here the recently developed machine-learning force-fields (MLFFs) have achieved a breakthrough as they have opened the possibility to achieve near first-principles accuracy MD trajectories of thousands of atoms on a nanosecond timescale that are needed to reliably model time-averaged NMR parameters.^63,64^ This allows for capturing cation dynamics and gaining detailed information about the microstructure in perovskites by MD simulation even for disordered systems such as mixed-cation or mixed halide perovskites,^26^ which are otherwise computationally prohibitively expensive.

In this work, we study the MA reorientational dynamics in mixed halide MAPbI_3−*x*_Br_*x*_ perovskites by a combined approach of experimental solid-state NMR spectroscopy and simulations based on MLFF molecular-dynamics. While the experimental data from both ^14^N NMR and ^1^H double-quantum (DQ) NMR spectroscopy indicate anisotropic dynamical reorientations due to preferred MA orientations depending on the halide composition and thus disorder in the inorganic sublattice, the MD data allow us to correlate these experimental results to restrictions of MA dynamics in certain local Pb_8_I_12−*n*_Br_*n*_ “cages”. Based on the experimental and simulated results, we develop a phenomenological model that correlates the ^1^H dipolar coupling and thus the MA dynamics with the local composition that reproduces the experimental data over the whole compositional range (*x* = 0–3). We show that the dominant interaction between the MA cations and the Pb–X lattice that influences the cation dynamics is the local electrostatic potential that is inhomogeneous in mixed halide systems. We expect that hydrogen bonds between the NH_3_ groups and the halides play a minor role in comparison. As such, we generate a fundamental understanding of the predominant interaction between the MA cations and the inorganic sublattice, as well as MA dynamics in asymmetric halide coordinations.

## 2. Experimental and computational details

### 2.1 Synthesis

We synthesized the perovskite powders employing a mechanochemical approach^65^ by ball milling in a Fritsch “Pulverisette 5/4” planetary ball mill. The reactants (MAI, MABr, PbI_2_, and PbBr_2_ or already completely synthesized hybrid perovskites) were weighed to the desired stoichiometry (see ESI† for exact amounts) and transferred into an 80 mL stabilized ZrO_2_ milling jar, containing stabilized ZrO_2_ milling balls with 10 mm diameter. Then, 9–11 mL cyclohexane was added as a milling agent. The powders were milled at 400 rpm for 5 min. Then milling was paused for 20 min to allow cooling of the jar. The procedure was repeated until the desired milling time was reached. The cyclohexane was evaporated subsequently by opening the finished milling jar and leaving it at room temperature in air for 15 minutes. Finally, we sieved the powder with a 63 or 90 μm sieve. MAI and MABr were synthesized as described in ref. 65, while PbI_2_ (purity >99.8%) and PbBr_2_ (purity >98%) were purchased from Sigma Aldrich and used as-is.

### 2.2 Powder X-ray diffraction

For powder diffraction analysis, samples were prepared inside a glovebox by hermetically sealing the powder in a 0.5 mm soda lime glass capillary. The X-ray diffractograms were recorded in capillary mode on a Panalytical Empyrean diffractometer using CuKα radiation and a PIXcel3D 1 × 1 detector.

### 2.3 Solid-state NMR spectroscopy

NMR spectra were recorded on Varian VNMRS systems operating at a magnetic field strength of 14.1 T (600 MHz) and 20.0 T (850 MHz). Probe heads used were a Varian 3.2 mm T3 HXY (600 MHz) and a Varian 1.2 mm T3 HXY (850 MHz) probe. The chemical shift was referenced using lead nitrate for ^207^Pb (−3494 ppm), ammonium chloride for ^14^N (−342.4 ppm) and adamantane for ^1^H (1.85 ppm) as secondary references. All experiments were performed at room temperature. Frame-cooling with boiled-off nitrogen was used to diminish frictional heating from MAS.

^207^Pb MAS NMR spectra were recorded at 5 kHz spinning speed using a spin-echo sequence (*ν*_1_(^207^Pb) ∼150 kHz) with a recycle delay of 0.5 s. The refocusing time was set as short as possible (10 μs) to diminish spin–spin relaxation effects. As the refocusing time of 10 μs is significantly shorter than the ^207^Pb spin spin (*T*_2_) relaxation in MAPbI_3_ of ∼40 μs^66^ or in MAPbBr_3_ (
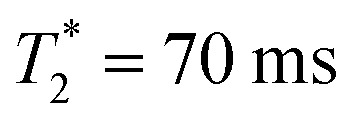
),^67^ the maximum change in relative intensity in the ^207^Pb NMR spectra due to *T*_2_ relaxation amounts to about 20% for high iodine coordinations, if there is a strong variation in *T*_2_ for the different [PbI_6−*n*_Br_*n*_]^4−^ sites in a mixed halide phase.

^14^N MAS NMR spectra were recorded using a solid echo sequence with a tip angle of 40° (*ν*_1_(^14^N) ∼56 kHz; refocusing time: one rotor period *ν*_rot_) at 10 kHz (mixed halide samples) and 5 kHz (MAPbI_3_, MAPbBr_3_) spinning speeds with a recycle delay of 0.5 s. All ^207^Pb and ^14^N NMR experiments were conducted at a magnetic field of 14.1 T.

High-resolution ^1^H MAS NMR experiments were performed at a magnetic field of 20 T (850 MHz) and a MAS frequency of 40 kHz. ^1^H DQ buildup curves were recorded using the BABA-xy16 (ref. 68) sequence (*ν*_1_(^1^H) ∼140 kHz). Zero-quantum (ZQ) reference measurements were used for normalization of the ^1^H DQ buildup curves:^68,69^1
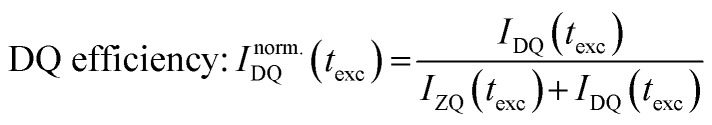


### 2.4 Machine-learning force-field molecular-dynamics simulations

A very similar approach as in ref. 26 was applied, which shows that this method is capable to describe MA_1−*x*_FA_*x*_PbI_3_ lattice dynamics. In short, a machine-learned potential energy surface was trained on-the-fly^63^ using the Gaussian approximation potential^70^ with two- and three-body descriptors and kernel function similar to the smooth overlap of atomic positions (SOAP) method.^71^ The descriptors are defined within a cutoff sphere of 6 and 4 Å for the two and three body term, respectively. This method is integrated in the Vienna *ab initio* simulation package (VASP).^72,73^ It calculates the potential energy, forces on the atoms, and stress tensor for all the reference structures, which are, by construction, well spread over the available structural phase space. The meta-gradient corrected functional SCAN^74^ is applied in the first-principles (FP) calculations, since it accurately describes the physical interactions in the material.^75^ The electronic minimization was performed within the projector augmented wave formalism^76^ with a plane wave basis (kinetic energy cutoff on the orbitals of 350 eV), a 2 × 2 × 2 k-point grid, and Gaussian smearing (*σ* = 0.01 eV). For the training, super cells consisting of 2 × 2 × 2 unit cells of MAPbI_3−*x*_Br_*x*_ were used: two for the ‘pure’ phases (*x* = 0, 3) and three for the mixed (*x* = 1.5) phase. These three comprise; one random mixed structure (R), the stable structure found by ref. 77 (A) and a maximally segregated (B) structure. This machine learning force field, capable to describe both ordered and random types of halide mixing, was then used (in production mode, *i.e.*, no more training) for all MD simulations shown in this work. The crystal structure database containing (DFT energies, forces and stresses) used to train the MLFFs can be accessed through 4TU.ResearchData.^78^

Isothermal and isobaric NPT-MD simulations of 4 × 4 × 4 super cells of MAPbI_3−*x*_Br_*x*_ were carried out with *x* = 0, 1.125, 1.5, 1.875 and 1, where Langevin thermo- and barostats were applied to control the conditions. The halides in the mixtures are randomly placed on high symmetry sites (see ESI for the ordered super cells†). All 4 × 4 × 4 cells were run for between 300 and 600 ps at 300 K under 1 bar standard pressure. We calculate the dipolar coupling coefficient for all H–H pairs and divide them in two groups, one for the intramolecular and one for intermolecular H–H connecting vectors. Applying ensemble averaging over time and space following the approach by Goc *et al.*^79–81^ then gives the average dipolar coupling that can be compared to the value obtained by NMR. Details of the computational methodology were presented in ref. 26 and apply here as well. How to relate the second moment calculations following the approach of Goc *et al.* to the dipolar coefficient extracted from the from double-quantum built-up curves following the approach by Saalwächter is presented in the ESI (Section 5.2†).

Details on *ab inito* EFG calculations from the MD trajectory of the *x* = 1.5 MAPbI_3−*x*_Br_*x*_ structure can be found in the ESI (Section 4†).

## 3. Results and discussion

We prepared single halide MAPbI_3_ and MAPbBr_3_, as well as mixed halide perovskites MAPbI_3−*x*_Br_*x*_ with *x* = 1, 1.5 and 2 by mechanochemical synthesis. The resulting powders were characterized by powder X-ray diffraction (PXRD) and ^207^Pb MAS NMR spectroscopy to determine their overall crystal structure, possible impurities, and the halide distribution over the inorganic sub-lattice.

**Fig. 1:**
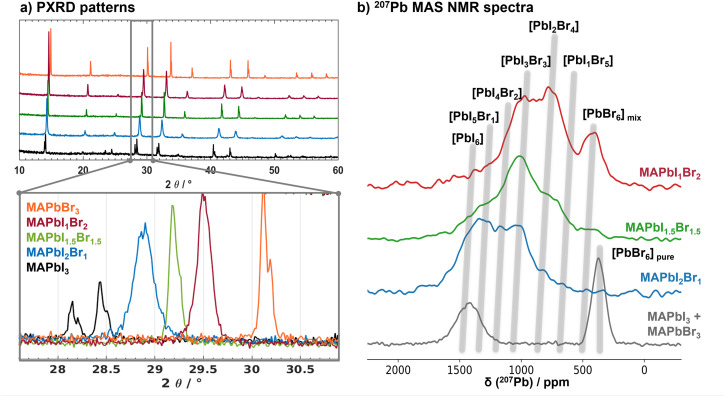
(a) PXRD patterns of MAPbI_3−*x*_Br_*x*_ (*x* = 0, 1, 1.5, 2, 3) revealing cubic perovskite lattices for all mixed samples and MAPbBr_3_, while MAPbI_3_ is in the tetragonal phase at room temperature. The zoom on the cubic 200 reflections between 27.5 and 31° 2*θ* highlights the sensitivity of the 2*θ* position (and the lattice constant) to the halide composition. (b) ^207^Pb MAS NMR spectra of a physical mixture of MAPbI_3_ and MAPbBr_3_, as well as the mixed halide perovskites MAPbI_3−*x*_Br_*x*_ (*x* = 1, 1.5, 2). The grey shaded areas indicate the chemical shifts for different [PbI_6−*x*_Br_*x*_] environments.

The PXRD pattern of MAPbI_3_ shows the tetragonal crystal phase at room temperature, while all mixed MAPbI_3−*x*_Br_*x*_ samples show reflections characteristic of a cubic crystal lattice (Fig. 1a). The corresponding cubic lattice constants linearly decrease from 6.27 to 5.93 Å with increasing Br^−^ content (Fig. S1†), reflected in the 2*θ* position of the 200 reflection as highlighted in the zoomed area of the PXRD patterns in Fig. 1a. The half width of the reflections is sensitive to the halide distribution. For the mixed halide samples the half width is close to those of the parent MAPbI_3_ and MAPbBr_3_ phases, indicating a homogenous distribution of the halides on the X-ray length scale. This result excludes a strong halide segregation and compositional fluctuations in large domains: our samples each consist of a single mixed halide perovskite phase with a single “average” crystal structure.

^207^Pb MAS NMR spectroscopy probes the local environment around the lead nuclei. The ^207^Pb isotropic chemical shift is very sensitive to the composition of the octahedral, (first) halide coordination shell.^45,49–51,66^Fig. 1b depicts the ^207^Pb NMR spectra of a physical MAPbI_3_/MAPbBr_3_ mixture (grey), as well as mixed MAPbI_2_Br_1_ (blue), MAPbI_1.5_Br_1.5_ (green) and MAPbI_1_Br_2_ (red). The average isotropic ^207^Pb chemical shifts, characteristic for the different [PbI_6−*n*_Br_*n*_]^4−^ coordinations, are indicated by grey lines. The slight increase (*i.e.* deshielding) with increasing iodine content is attributed to the increase in lattice constants with increasing iodine content.^50^ While for MAPbI_2_Br_1_ and MAPbI_1.5_Br_1.5_ the [PbI_6−*n*_Br_*n*_]^4−^ signal integrals fit the values expected for a random halide distribution characteristic for solid solutions with a deviation of less than 5% (Fig. S2 and Tables S2–S4†), in the case of MAPbI_1_Br_2_ the population of [PbBr_6_]^4−^ environments is roughly 7% higher and the population of [PbI_1_Br_5_]^4−^ about 11% lower than expected for a random distribution. Hence, we observe a small tendency for anion ordering for (only) the MAPbI_1_Br_2_ composition. This can only give rise to composition fluctuations on short length scales as the PXRD patterns reveal that all samples are single phase.

These mixed halide samples are the basis for investigating the influence of the inorganic Pb–X sublattice on the organic MA cation dynamics and especially the impact of halide mixing. We used a combination of ^14^N MAS NMR and ^1^H DQ NMR spectroscopy to extract the average ^14^N quadrupolar interaction and the average ^1^H dipolar coupling of the MA cations. Both quantities probe different anisotropic interactions, *i.e.*, the quadrupolar and the dipolar interaction, respectively, but both, however, are sensitive to the reorientational dynamics of the MA cations.^26–29,55^

**Fig. 2:**
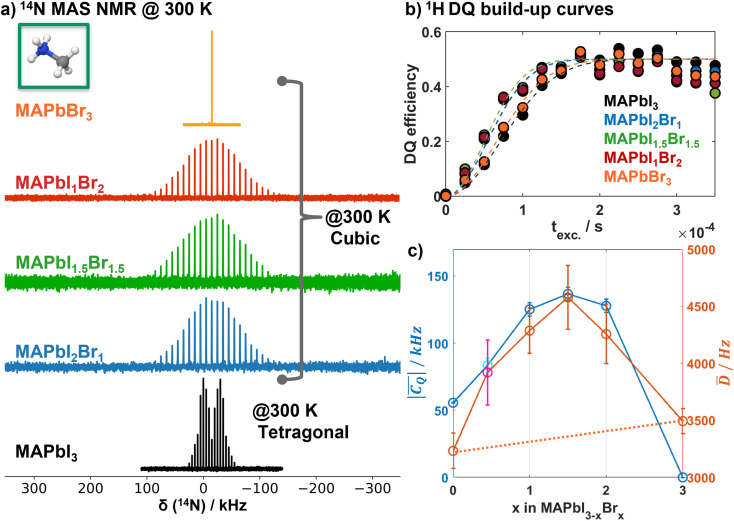
(a) ^14^N MAS NMR spectra of MAPbI_3−*x*_Br_*x*_ (*x* = 0, 1, 1.5, 2, 3) recorded at spinning speeds of 5 kHz (*x* = 0, 3) and 10 kHz (*x* = 1, 1.5, 2). Fits of the spectra to extract the average quadrupolar coupling constants are depicted in Fig. S3.† (b) Normalized ^1^H DQ build-up curves of MAPbI_3−*x*_Br_*x*_ (*x* = 0, 1, 1.5, 2, 3) and corresponding fits (dashed lines) following the approach of Saalwächter *et al.*^68,69^ to extract the average dipolar coupling constants. (c) Average quadrupolar coupling 
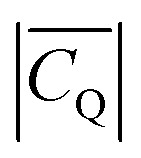
 (blue) and average dipolar coupling *D̄* (orange) as a function of Br content *x* in MAPbI_3−*x*_Br_*x*._ The data points (cyan; magenta) at *x* = 0.45 corresponds to a double-mixed MA_0.15_FA_0.85_PbI_2.55_Br_0.45_ composition with additional MA/FA mixing on the A site, which was previously analyzed with respect to the ^1^H dipolar coupling,^26^ while the corresponding ^14^N MAS spectrum is depicted in Fig. S4.† Both, the average quadrupolar and the average dipolar coupling, show a parabolic behavior as a function of halide composition.

As NMR probes a time-average of the anisotropic ^14^N quadrupolar interaction it provides detailed information on cation reorientation, *i.e.*, the phase space visited by the cation's atomic coordinates during the NMR measurement.^28,29,44,55,66^ For cubic perovskite phases a fast and isotropic MA reorientation is expected, resulting in a single isotropic resonance due to complete averaging of the quadrupolar coupling (
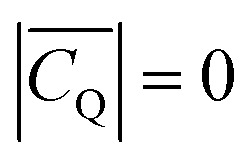
), while, *e.g.*, tetragonal MAPbI_3_ phase exhibits a spectrum indicative of an axially symmetric quadrupolar interaction (non-zero electric field gradient, 
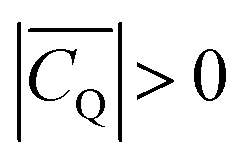
, *η* = 0).^66^ This is reflected in the experimental ^14^N room temperature MAS NMR spectrum of MAPbI_3_ (Fig. 2a), which exhibits the typical *η* = 0 quadrupolar spinning sideband manifold corresponding to a quadrupolar coupling constant 
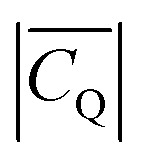
 of ∼56 kHz. The ^14^N NMR spectrum of cubic MAPbI_3_,^66^ and MAPbBr_3_ (Fig. 2a) shows no quadrupole coupling (
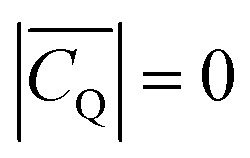
): the fast reorientation of the MA cations makes that the ^14^N nucleus, on average, sees a cubic crystal field as previously observed.^27,28^ In contrast, the ^14^N MAS NMR spectra of the cubic mixed halide MAPbI_3−*x*_Br_*x*_ perovskites do exhibit a clear quadrupolar interaction (Fig. 2a, 
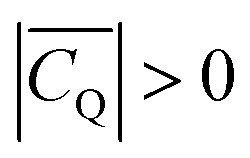
). The Gaussian-like signal shape indicates disorder in the halide distribution in the immediate surroundings of the MA cations and/or restrictions on MA reorientations, or a superposition thereof.

The ^14^N MAS NMR spectra are analysed using the Czjzek^82,83^ model to describe the electrical field gradient distribution in the disordered mixed halide compositions (Fig. S3–S5 and Table S5†). In this way, the average quadrupolar coupling 
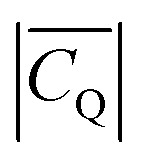
 is extracted for all mixed halide compositions. Starting from tetragonal MAPbI_3_ it increases for the cubic mixed halide phases with increasing Br content *x* until a maximum average 
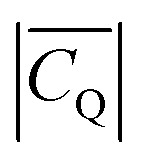
 of ∼137 kHz for *x* = 1.5 (MAPbI_1.5_Br_1.5_) is reached (Fig. 2c, blue). Higher Br contents (*x* > 1.5) lead to a reduction of the average 
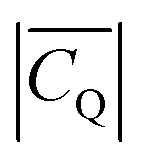
 resulting in a parabolic-like behaviour of the average 
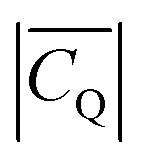
 as a function of Br content *x* (Fig. 2c, blue). We also included a double-mixed MA_0.15_FA_0.85_PbI_2.55_Br_0.45_ composition, which we previously investigated with respect to the MA/FA distribution by a combination of ^1^H DQ NMR spectroscopy and MLFF MD simulations.^26^ The ^14^N MAS NMR spectrum (Fig. S4†) consists of two sets of spinning sideband manifolds corresponding to the MA and FA cations. The Czjzek refinement of the spectrum revealed an average 
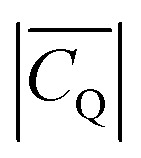
 of ∼84 kHz for the MA cations, which corroborates the observed parabolic behavior of pure MA compositions.

The ^1^H–^1^H dipolar coupling is also sensitive to the MA cation environment and dynamics. Hence we measured the ^1^H DQ build-up curves for different MAPbI_3−*x*_Br_*x*_ compositions (*x* = 0, 1, 1.5, 2 and 3) and applied a similar analysis as in ref. 26 to extract the average ^1^H–^1^H dipolar interactions. Interestingly, the average ^1^H dipolar coupling *D̄* (Fig. 2c) displays a similar parabolic behaviour as the ^14^N average 
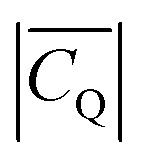
. It increases from ∼3200 Hz for pure MAPbI_3_ to a maximum of ∼4600 Hz for MAPbI_1.5_Br_1.5_. With higher Br contents *D̄* decreases again to ∼3490 Hz for MAPbBr_3_. Although the individual data points in our 
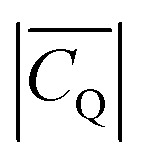
 and *D̄* determinations do have a substantial error bar (Fig. 2c), it is clear that all the data closely follow a parabolic trend depending on the halogen composition thus supporting our analysis.

In ref. 26 we investigated the influence of cation reorientation on the average ^1^H dipolar couplings for MA_1−*y*_FA_*y*_PbI_3_ systems, *i.e.*, with varying amount of different cations instead of anions. In these cubic systems the MA and FA motions result in a complete averaging of the intramolecular contributions to the ^1^H–^1^H dipolar couplings. Hence the experimental ^1^H dipolar couplings are determined by the inter-molecular dipolar contributions only. These are dependent on the number of contributing ^1^H spins and the distance between the cations, and as such the lattice constant.^26^ Turning back to MAPbI_3−*x*_Br_*x*_, if only the inter-molecular dipolar terms are relevant, we would expect a linear increase of the ^1^H dipolar couplings (from 3200 to 3490 Hz) with increasing Br content *x*, following the linear trend of lattice contraction (Fig. S1†). This is not the parabolic behaviour that we observe, however. Hence, intra-molecular dipolar terms/contributions are no longer fully averaged for mixed halide compositions, which must be linked to a change of the cation reorientational mobility either due to a restriction in spatial degrees of freedom or to a very significant reduction in reorientation frequency.

To understand the nature of the parabolic behaviour of the ^14^N quadrupolar and ^1^H dipolar couplings, it is necessary to investigate the interaction between the inorganic Pb–X sublattice and the organic MA cations and its impact on the MA dynamics. Therefore, we have carried out molecular-dynamics (MD) simulations for several MAPbI_3−*x*_Br_*x*_ compositions. For this we used a trained machine learning force field (MLFF) with near first-principles accuracy. Details can be found in Section 2 and the ESI.† To account for the large possible variety of anion arrangements on the lattice the force field training was carried out on supercells with *x* = 0, 1.5 and 3 and, for *x* = 1.5, on a randomly mixed structure (R), the stable structure found by ref. 77 (A) and a maximally segregated (B) structure, *i.e.* alternating layers of I^−^ and Br^−^. The MD calculations were carried out for systems with *x* = 0, 1.125, 1.5, 1.875 and 3 at 300 K.

**Fig. 3:**
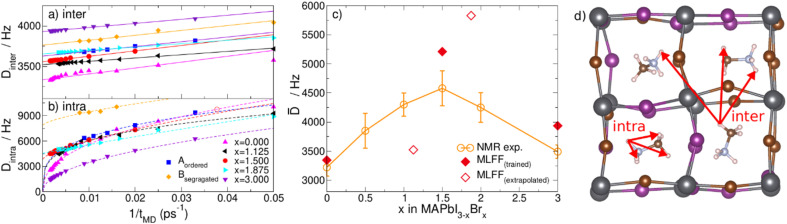
MLFF modelling of the average dipolar coupling. The (a) inter-molecular and (b) intra-molecular contribution to the total ^1^H–^1^H dipolar coupling as function of the inverse MD simulation time for the different MAPbI_3−*x*_Br_*x*_ systems. Dashed lines are fit functions and extrapolate to infinite simulation time. (c) The total average dipolar coupling as function of x with randomly arranged halides (red) compared to the experimental NMR data (orange). (d) Illustration of the intra- and inter-molecular H–H connection vectors in the perovskite lattice.

Fig. 3 shows the MLFF simulated inter- and intramolecular contributions to the ^1^H–^1^H dipolar coupling as function of the inverse simulation time (1/*t*_MD_) for various *x*. These are calculated as time averages over the MD trajectories.^26^ As practically infinite simulation times would be necessary for a comparison to the experimental NMR dipolar couplings, the inter- and intra-molecular averages are extrapolated (1/*t*_MD_ → 0). Fig. 3a shows that the intermolecular contributions behave very similar in all systems, and that the limit of infinite simulation time is easily obtained by linear extrapolation. The situation is more complicated for the intra-molecular contributions depicted in Fig. 3b. The intra-molecular time-averages all display a variation that is well-described by a root-function, 
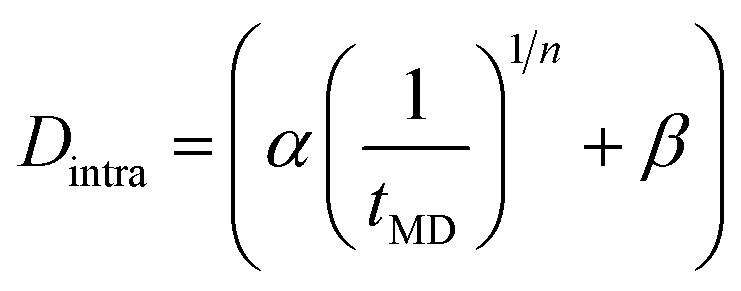
. Here *n* and *β* are fit parameters, where *β* represents the intra-molecular contribution at infinite simulation time, *i.e.*, the *D*_intra_ we need to simulate the experimental dipolar couplings. Both the *x* = 0 and *x* = 3 system are well-fitted by a negligible *β* and *n* = 3 and 2, respectively. However, in case of 0 < *x* < 3 a finite intra-molecular dipolar contribution *β* remains. In case of the ordered halide arrangements (structures A & B), extrapolated intramolecular dipolar term *β* is much larger than in the random mixture. Using the extrapolated *D̄*_inter_ and *D̄*_intra_ contributions, the total average dipolar coupling can be calculated as:
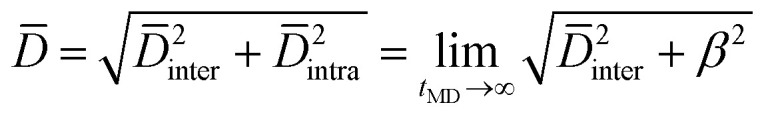


The calculated total dipolar couplings of 3634 and 8760 Hz for the ordered structures A and B, respectively, are much lower and higher than the experimental value of ∼4600 Hz, which serves as supporting evidence that the halides are indeed randomly mixed. Fig. 3c shows the calculated *D̄* for the random halide mixtures (red diamonds) compared to the NMR data (orange line) of Fig. 2c. The agreement between NMR and MD is very good for *x* = 0, 1.5, 3, *i.e.*, the compositions on which the MLFF has been explicitly trained. The discrepancies around *x* = 1.125 and *x* = 1.875 (open red diamonds) coincide with those compositions for which we allow the MLFF to extrapolate and were not included in the training. The erroneous predictions could be related to either (a) the simulation cell size, which might be too small to well represent random statistics at these values of *x*, or (b) the quality of the MLFF that might not interpolate sufficiently accurate for these compositions. In the following we focus on *x* = 0, 1.5 and 3 for which these limitations do not apply.

**Fig. 4:**
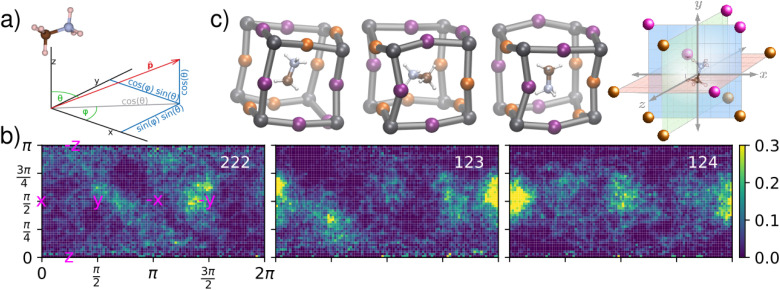
Effect of local inhomogeneous halide environment in the random *x* = 1.5 system on orientational dynamics of the MA cation. (a) The molecular C–N axes of all molecules are expressed in polar coordinates {*θ*, *ϕ*}. (b) Polar distributions of cations in three different environments shown by the insets in (c). The number of iodines in each of the three planes is used to characterize the local ‘cage’.

We further analyse the MD trajectories to understand how *β* is determined by the MA reorientational dynamics. The trajectories yield the time evolution of the C–N vector *p*_*i*_(*t*) of each individual MA cation *i* (1 ≤ *i* ≤ 64). The orientation of the C–N axis is expressed in spherical coordinates {*θ*, *ϕ*} as shown in Fig. 4a. For each cation we make a polar distribution showing its orientations during the complete MD simulation. At 300 K, the pure bromine phase (*x* = 3) shows similar isotropic polar distributions for all MA molecules.^42^ The pure iodine phase (*x* = 0) still has a small tetragonal distortion at this temperature resulting in a slight preference for face-diagonal orientations on top of a isotropic distribution.^63^ This picture significantly changes upon mixing the halides on the inorganic perovskite sub-lattice (0 < *x* < 3). For all mixed halide structures, both random and ordered, as well as for all investigated halide compositions *x*, we observe some flat and a suite of distributions with hot spots. This is illustrated in Fig. 4b for three MA cations of the *x* = 1.5 randomly mixed structure (see ESI for all polar distributions, Fig. S15–S17†). The selected cations correspond to the least (left) and most (middle and right) corrugated polar distributions. Firstly, we observe that on the local scale of a single cation cubic symmetry is broken. The global cubic symmetry is recovered on the large length scale as the average over many random cells. Secondly, we observe preferential orientations of the MA cations that correlate to the local halide ‘cage’ in which they are confined. The cages corresponding to the three distributions are shown in Fig. 4c.

**Fig. 5:**
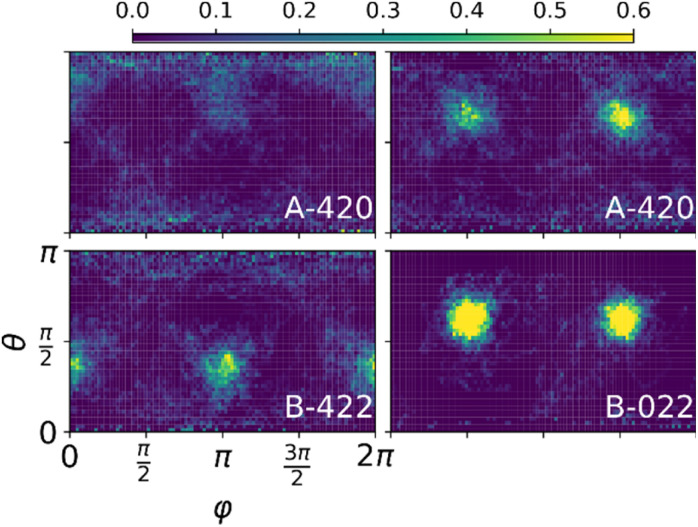
Polar distributions of MA cations in the ordered A and B structures indicating more preferred orientations compared to the random structure. Note that the range in the color scale is double that of Fig. 4b.

In order to analyse whether the dynamics of the molecule is set by the first shell of halides we characterize these local environments in two ways: (i) by their local concentration (*x*_i_) and (ii) by three numbers, indicating the number of iodines in each of the planes bisecting the cube as sketched in Fig. 4c. As a measure for the corrugation of the polar distributions we calculate the variance (*σ*^P^_i_) of each of them. Fig. S18–S20† show that the local concentration *x*_i_ is not correlated with *σ*^P^_i_. This can also be seen in Fig. 4b where the left and middle distributions correspond to cations in different cages, but with the same fraction *x*_i_ = 1.5. The cage [222] on the left has two iodines in each plane, while the middle cage [123] has only one iodine in the *xy* plane and three in the *yz* plane. The right cage [124] shows an even higher level of corrugation (*σ*^P^_i_) than the middle one. Its local symmetry deviates most from cubic and, resultingly, shows the highest preference of the cation for a specific orientation. In Fig. S18–S20† we show that this is not an anomaly, but that there is a small but statistically significant level of correlation between the level of cubic symmetry breaking (*σ*^SB^_i_) and the measured corrugation (*σ*^P^_i_). To calculate *σ*^SB^_i_ we simply compute the variance of the three numbers [*XYZ*] describing the local environment. The modest Pearson correlation coefficient (∼0.3) indicates that the cation orientations are definitely influenced by the layout of the halide species on the cage forming the first coordination shell but that they are not completely determined by it. The picture becomes more pronounced when order is introduced in the halide mixture as tested by structures A & B. In Fig. 5 two representative polar plots of cations in structures A and B are very corrugated and indicate strongly preferred orientations in the B structure and slightly less preferred (but more than in the random structure) orientations in structure A. The polar distributions for all 64 molecules in these systems are shown in Fig. S13 and S14.† The ordered B system with maximally segregated halides even shows half of the cations pinned.

Let us summarize our findings up to this point. The NMR experiments indicate a random mixing of the halides. From the simulations we infer that inhomogeneous cage configurations (larger *σ*^SB^_i_) that lack inversion symmetry have a higher probability to display preferred cation orientations. These cations will contribute *D̄*_intra_ > 0, in contrast to the cations in the pure phases (*x* = 0 and 3) for which *D̄*_intra_ is negligible. Now, combinatorics tells us that in case of random mixing the chance of finding a local cage with a large *σ*^SB^_i_ value is maximal at *x* = 1.5 and decreases to zero when going to *x* = 0 or 3. Consequently, *D̄*_intra_ as function of *x* should have the same maximum and minima. We would like to stress that the increase of *D̄*_intra_ is not the result of cations that do not reorient. Almost all of them show a slow (*τ* between 3 and 25 ps) and a fast (0–3 ps) decorrelation process, see ESI Fig. S21–S24,† both much faster than the NMR timescale. Solely in case of the halide ordered B structure, half of the cations do not decorrelate within the timeframe of the MD simulation.

**Fig. 6:**
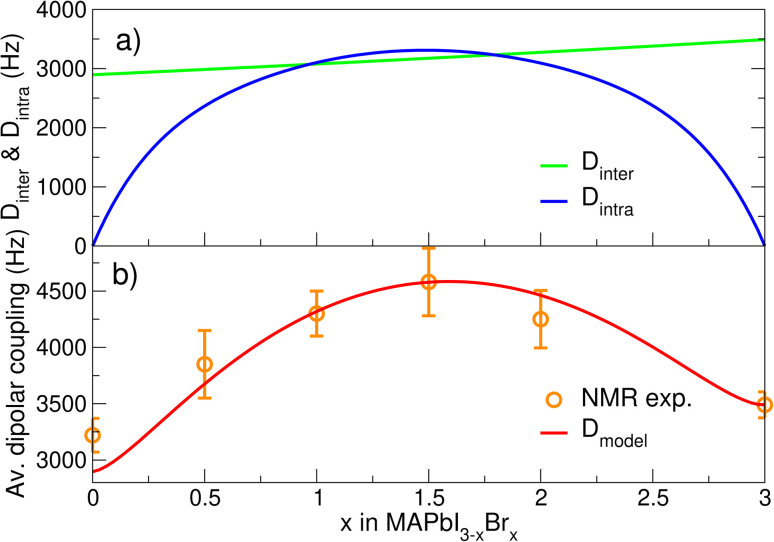
Phenomenological model of the average dipolar coupling. (a) The coupling as function of *x* can be decomposed in a *D*_inter_ and a *D*_intra_ contribution. (b) Model of the average dipolar coupling (red) and the experimental NMR dipolar couplings (orange).

Inspired by the above observations, we now develop a simple phenomenological model for *D̄* as a function of halide composition *x*. In ESI Section 5.5† we show that the average *σ*^SB^_i_ value in an infinite random ensemble with concentration *x* closely resembles the parabola curve of the mixing entropy of a binary gas. We therefore model the intra contribution to the average ^1^H–^1^H dipolar coupling simply as *D*^m^_intra_(*x*′) = −*m*_1_(*x*′ln(*x*′) + (1 − *x*′)ln(1 − *x*′)), where *x*′ = *x*/3, resulting in the parabola-like shape in Fig. 6a. For the linear inter-molecular contribution, the effect of the lattice expansion with *x* is included as 
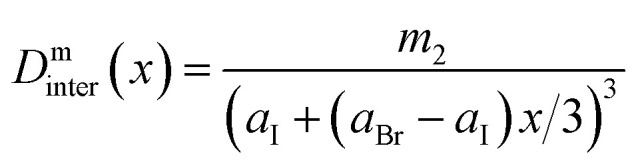
. Here *a*_Br_ and *a*_I_ are the (pseudo)-cubic lattice constants of pure Br and I (Table S1†). The total dipolar coupling can then be calculated as, 
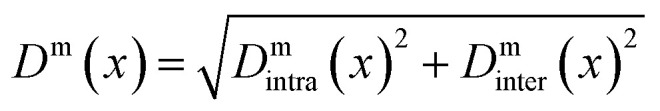
. The model fit parameters *m*_1_ = 4760 and *m*_2_ = 3490/*a*_Br_^3^ are determined by the requirement to reproduce the NMR data at *x* = 1.5 and 3. The agreement between the model and the NMR data as shown in Fig. 6b is remarkable, which allows a prediction of the average MA restriction solely based on the halide cage disorder. In Fig. 6a we further observe that the intra-molecular contribution becomes even larger than the inter contribution. This is remarkable as the cations still reorient much faster than the NMR timescale. However, as we have shown here, they exhibit preferential orientations related to the local cubic symmetry breaking in the random mix. In Fig. 2c a similar parabolic trend as for *D*^m^_intra_ was observed for the experimental ^14^N quadrupolar interactions. The fact that we see only a partial averaging of the ^14^N quadrupolar coupling and a distribution thereof (reflected by the gaussian shape, Fig. 2a) further supports that the local halide configurations indeed enhance preferential MA orientations. This is also reflected in EFG calculations of the average quadrupolar coupling 
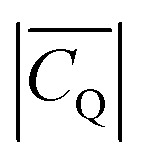
 from the MD trajectories of the *x* = 1.5 structure (ESI Section 4†), where the calculated average 
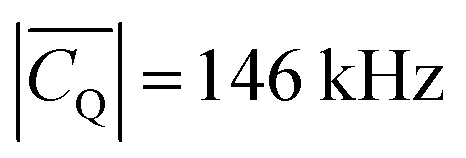
 is very close to the experimental value of 137 kHz. This is considerable, but much smaller than the number for static cations: for the 64 cations in a single MD timeframe we calculate an average 
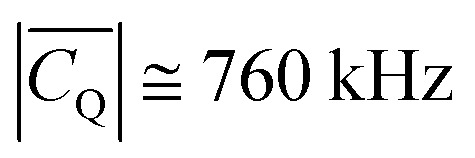
. While changes in the MA reorientation rates upon random halide mixing were observed earlier,^31,35^ our analysis demonstrates the physical effect of the local inhomogeneity of Pb_8_X_12_ cages causing rather an asymmetry in MA orientation instead of a significant reduction of the reorientation rates as shown in the MD trajectories (see ESI Section 5.8†).

The phenomenon of the preferred MA orientations in asymmetrically mixed halide environments may be caused by various interactions between the MA cations and the inorganic Pb–X sublattice, such as hydrogen bonding, steric effects, or the electrostatic potential. Hydrogen bonds result in a downfield shift (higher ppm values) of the ^1^H NMR signal of the involved group due to the deshielding of H atoms. The ^1^H chemical shift is thus directly correlated to the hydrogen bond strength.^84,85^ The difference in ^1^H chemical shifts for the NH_3_^+^ group of MAPbI_3_ and MAPbBr_3_ is as small as 0.15 ppm (Fig. S6†) indicating very similar hydrogen bond strengths for I and Br in hybrid lead halide perovskites. This is also observed for the mixed halide composition MAPbI_1.5_Br_1.5_ (Fig. S6†). Furthermore, we do not observe significant differences in the polar distributions for *e.g.*, [111] and [222] configurations indicating that the geometry of the halide configuration in the A cage plays a minor role. As strong hydrogen bonds require a geometry fit between the NH_3_ group and the specific halide configurations in addition to the minor differences in the experimental ^1^H chemical shifts, we conclude that hydrogen bonds or differences in hydrogen bond strength are not causing preferred MA orientations in the mixed halide perovskites. Steric effects due to differences in the ionic radii of I^−^ and Br^−^ (Δ ∼0.24 Å),^86^ also do not seem very plausible as similar MA decorrelation times are observed for MAPbI_3_ and MAPbBr_3_.^42,63^ Therefore, we qualitatively explain the existence of preferred MA orientations by the difference in electronegativity of the Br and I ions. The differences in the electronegative potential result in “sinks” within the PbX_12_ cage where the NH_3_ groups preferably reside. Here, we stress that the potential difference is the important factor, which also causes the parabolic shape. However, as seen from our MD analysis the effect of MA orientations is not solely correlated to the local cage configurations but goes beyond.

## 4. Conclusion

The combined NMR spectroscopic and MD simulation study of mixed halide perovskite MAPbI_3−*x*_Br_*x*_ has conclusively shown that at ambient temperature, pressure and under dark conditions iodine and bromine ions are randomly distributed over the MAPbX_3_ sublattice. Hence there is a large variation in how the halides are distributed over the inorganic cages that confine the individual MA cations. We demonstrated that the resulting inhomogeneity of the cages gives rise to preferential MA orientations in their individual cage.

The variation of the halide environment of the MA cations depends on the Br fraction *x*. At *x* = 1.5 there is the largest possible variation of local cage environments with local symmetry breaking, whereas at *x* = 0 and 3 there is no variation. The level of local symmetry breaking, *i.e.*, deviation from cubic symmetry, and thus how the I^−^ and Br^−^ anions are distributed over the 12 sites of the inorganic cage matters, as shown by MLFF MD simulations. Depending on the local cage type, a MA cation can (almost) isotropically reorient or be constrained to spend more time in certain preferred direction(s) while it tumbles on-site. We have developed a phenomenological model for random halide distributions wherein we link the ^1^H–^1^H dipolar coupling and ^14^N quadrupolar interactions to the local halide surrounding of the cations. It describes with good agreement the experimental dipolar coupling for all compositions *x*. We distinguish between inter-molecular contributions that scale according to the global lattice constants, and intra-molecular contributions that scale with the spread in possible local symmetry broken environments that can occur in a random mixture of composition *x*. The dominant physical effect is attributed to the random mixture of the halides and their difference in electronegativity leading to local variations in anisotropic electrostatic potentials rather than variations in local hydrogen bonding. The dynamics of the cation is directly influenced by the layout of the twelve nearest halides composing the A cage, but this does not fully determine it. The composition of neighbouring cages affects the cation dynamics as well.

As the restriction in MA orientation directly correlates with the halide distribution in the inorganic sub-lattice, we expect that the preferential cation orientations are also randomly distributed on the cation sublattice. In this case, we do not expect a collective behaviour resulting in dipole alignment, *i.e.*, we do not expect a macroscopic polarization. However, during illumination, light-induced halide segregation may cause some degree of halide ordering which potentially results in a collective cation behaviour and/or alignment. This is supported by our results for the halide ordered structures, where the cation dynamics are maximally restricted. If such collective behaviour upon illumination occurs, and if it results in partial dipole alignment it might result in an electrostatic field and thus facilitate charge carrier separation and lead to longer charge carrier lifetimes. The interplay between halide migration, an(isotropic) cation reorientation and charge separation and dynamics is very complex and will be an important subject for future research. As under dark conditions, random halide arrangements are thermodynamically stable,^17,87,88^ it is currently challenging to study local structural and dynamical features for both the halides and the MA cations, in more ordered mixed halide configurations, which occur under illumination. Therefore, it will be a future task to develop NMR setups and techniques, which allow for a homogenous illumination *in situ* to link halide migration and an(isotropic) cation reorientation. This may allow for developing design strategies for photostable mixed halide perovskites.

## Conflicts of interest

There are no conflicts to declare.

## Supplementary Material

TA-011-D2TA09069D-s001

TA-011-D2TA09069D-s002

TA-011-D2TA09069D-s003

TA-011-D2TA09069D-s004

TA-011-D2TA09069D-s005
