# Supplementary material for: Disorder to order: how halide mixing in MAPbI3−xBrx perovskites restricts MA dynamics
Source: J Mater Chem A Mater. 2023 Feb 1;11(9):4587–97. doi: 10.1039/d2ta09069d (PMC10294545; doi:10.1039/d2ta09069d)
Supplement: TA-011-D2TA09069D-s001 [file TA-011-D2TA09069D-s001.pdf]

Electronic Supplementary Material (ESI) for Journal of Materials Chemistry A.
This journal is © The Royal Society of Chemistry 2023

Supporting Information for

Disorder to order: how halide mixing in $MAPbI_{3-x}Br_x$ perovskites restricts MA dynamics

Kostas Fykouras[1], Jonathan Lahnsteiner[1], Nico Leupold[2], Paul Tinnemans[3], Ralf Moos[2], Fabian Panzer[4], Gilles A. de Wijs[3], Menno Bokdam[1]* and Helen Grüninger[3,5]* and Arno P.M. Kentgens[3]

[1] Faculty of Science and Technology and MESA+ Institute for Nanotechnology, University of Twente, P.O. Box 217, 7500 AE Enschede, The Netherlands.

[2] Department of Functional Materials, University of Bayreuth, Universitätsstraße 30, 95447 Bayreuth, Germany.

[3] Institute for Molecules and Materials, Radboud University, Heyendaalseweg 135, 6525 AJ Nijmegen, The Netherlands.

[4] Soft Matter Optoelectronics, University of Bayreuth, Universitätsstraße 30, 95447 Bayreuth, Germany.

[5] Inorganic Chemistry III and Northern Bavarian NMR Centre, University of Bayreuth, Universitätsstraße 30, 95447 Bayreuth, Germany.

Corresponding authors: m.bokdam@utwente.nl; helen.grueninger@uni-bayreuth.de

1. Exact amounts of precursors for mechanochemical synthesis

$MAPbI_3$: 1.900 g of MAI (11.95 mmol) and 5.509 g of PbI_2 (11.95 mmol) were milled for 50 min to prepare the black powder.

$MAPbI_2Br_1$: 5.150 g $MAPbI_3$ (8.35 mmol) and 2.000 g $MAPbBr_3$ (4.18 mmol) were milled for 50 min to prepare the black powder.

$MAPbI_{1.5}Br_{1.5}$: 0.867 g MAI (5.45 mmol), 0.610 g MABr (5.45 mmol), 2.512 g PbI_2 (5.45 mmol) and 2.000 g $PbBr_2$ (5.45 mmol) were milled for 80 min to prepare the black powder.

$MAPbI_1Br_2$: 1.733 g MAI (10.90 mmol) and 4.000 g $PbBr_2$ (10.90 mmol) were milled for 80 min to prepare the red powder.

$MAPbBr_3$: 1.525 g of MABr (13.62 mmol) and 5.000 g of $PbBr_2$ (13.62 mmol) were milled for 80 min to prepare the orange powder.

2. Additional PXRD data

Table S1. Experimental lattice constants for $MAPbI_{3-x}Br_x$ (x= 0, 1, 1.5, 2, 3) extracted by refinements of the PXRD patterns depicted in Fig. 1a.

	Lattice constant a / Å	
**$MAPbI_3$**	6.27	Modelled as pseudo-cubic
**$MAPbI_2Br_1$**	6.17	
**$MAPbI_{1.5}Br_{1.5}$**	6.11	
**$MAPbI_1Br_2$**	6.04	
**$MAPbBr_3$**	5.93	

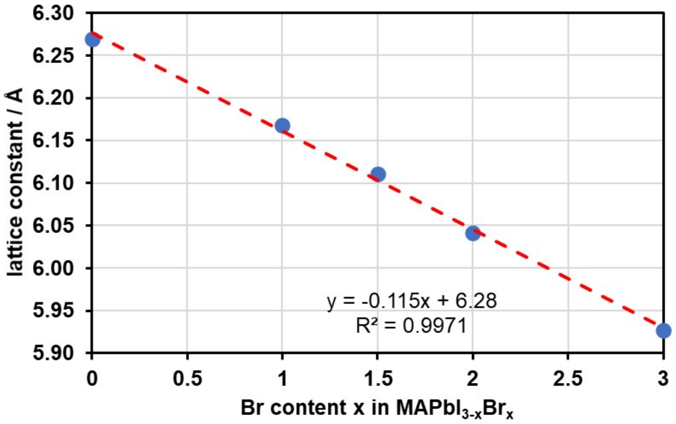

Figure S1. Experimental lattice constant a (cubic lattice) as a function of Br content x in $MAPbI_{3-x}Br_x$ (blue dots) revealing a linear trend (red dotted line). The equation of the linear fit is given in the figure.

3. Additional solid-state NMR spectroscopic data

3.1. ^{207}Pb NMR spectroscopy

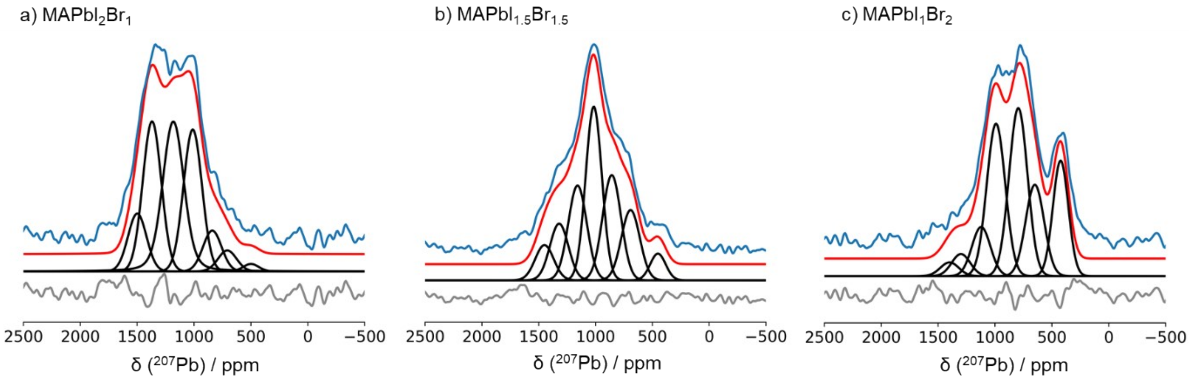

Figure S2. Experimental ^{207}Pb MAS NMR spectra (blue), corresponding deconvolutions (red) using seven resonances corresponding to the different $PbI_{6-x}Br_x$ environments (x=0, 1, …, 6: black) and the difference between experiment and deconvolution (grey) of $MAPbI_2Br_1$ (a), $MAPbI_{1.5}Br_{1.5}$ (b) and $MAPbI_1Br_2$ (c). The chemical shifts for the $PbI_{6-x}Br_x$ and the corresponding populations resulting from the fit are summarized in Tab. S2 to S4. As literature data[1–3] show CSA effects are minor and where therefore neglected for the fits.

As the refocusing time of 10 μs used for the ^{207}Pb spin echo experiments (Fig 2 and Fig S2) is significantly shorter than the ^{207}Pb spin spin (T_2) relaxation determined for $MAPbI_3$ of ~40 μs[4] or in $MAPbBr_3$ (T_2*=70 ms)[5], we do not expect significant changes in the ^{207}Pb NMR spectra due to T_2 relaxation. Thus, we evaluate the ^{207}Pb spectra as pseudo-quantitative and compare the experimental integrals of $PbI_{6-x}Br_x$ environments (x=0, 1, …, 6) to the ones expected from the statistics for a random distribution of halides (Tab. S2-S4). This is supported by the fact that the halide composition obtained from XRD data matches those obtained from the NMR spectra for each halide composition.

Table S2. ^{207}Pb chemical shift and resulting normalized integrals for each of the seven fitted $PbI_{6-x}Br_x$ resonances in Fig. S2a, as well as the corresponding theoretical probabilities for the case of a random halide distribution (calculated using a binomial function) in $MAPbI_2Br_1$.

Site	Shift / ppm	Experimental integrals	Theoretical integrals for a random dist.	Difference
**[PbI_6]**	1500	10	9	1
**[PbI_5Br_1]**	1370	25	27	-2
**[PbI_4Br_2]**	1180	31	33	-2
**[PbI_3Br_3]**	1010	23	22	1
**[PbI_2Br_4]**	860	7	8	-1
**[PbI_1Br_5]**	710	3	2	1
**[$PbBr_6$]**	500	1	0	1

Table S3. ^{207}Pb chemical shift and resulting normalized integrals for each of the seven fitted $PbI_{6-x}Br_x$ resonances in Fig. S2b, as well as the corresponding theoretical probabilities for the case of a random halide distribution (calculated using a binomial function) in $MAPbI_{1.5}Br_{1.5}$.

Site	Shift / ppm	Experimental integrals	Theoretical integrals for a random dist.	Difference
**[PbI_6]**	1450	7	2	5
**[PbI_5Br_1]**	1320	11	9	2
**[PbI_4Br_2]**	1160	18	23	-5
**[PbI_3Br_3]**	1000	28	31	-3
**[PbI_2Br_4]**	850	20	23	-3
**[PbI_1Br_5]**	690	13	9	4
**[$PbBr_6$]**	450	4	2	2

Table S4. ^{207}Pb chemical shift and resulting normalized integrals for each of the seven fitted $PbI_{6-x}Br_x$ resonances in Fig. S2c, as well as the corresponding theoretical probabilities for the case of a random halide distribution (calculated using a binomial function) in $MAPbI_1Br_2$.

Site	Shift / ppm	Experimental integrals	Theoretical integrals for a random dist.	Difference
**[PbI_6]**	1400	2	0	2
**[PbI_5Br_1]**	1300	4	2	2
**[PbI_4Br_2]**	1120	8	9	-1
**[PbI_3Br_3]**	990	26	23	3
**[PbI_2Br_4]**	800	29	34	-5
**[PbI_1Br_5]**	650	16	27	-11
**[$PbBr_6$]**	420	16	9	7

3.2. ^{14}N MAS NMR spectroscopy

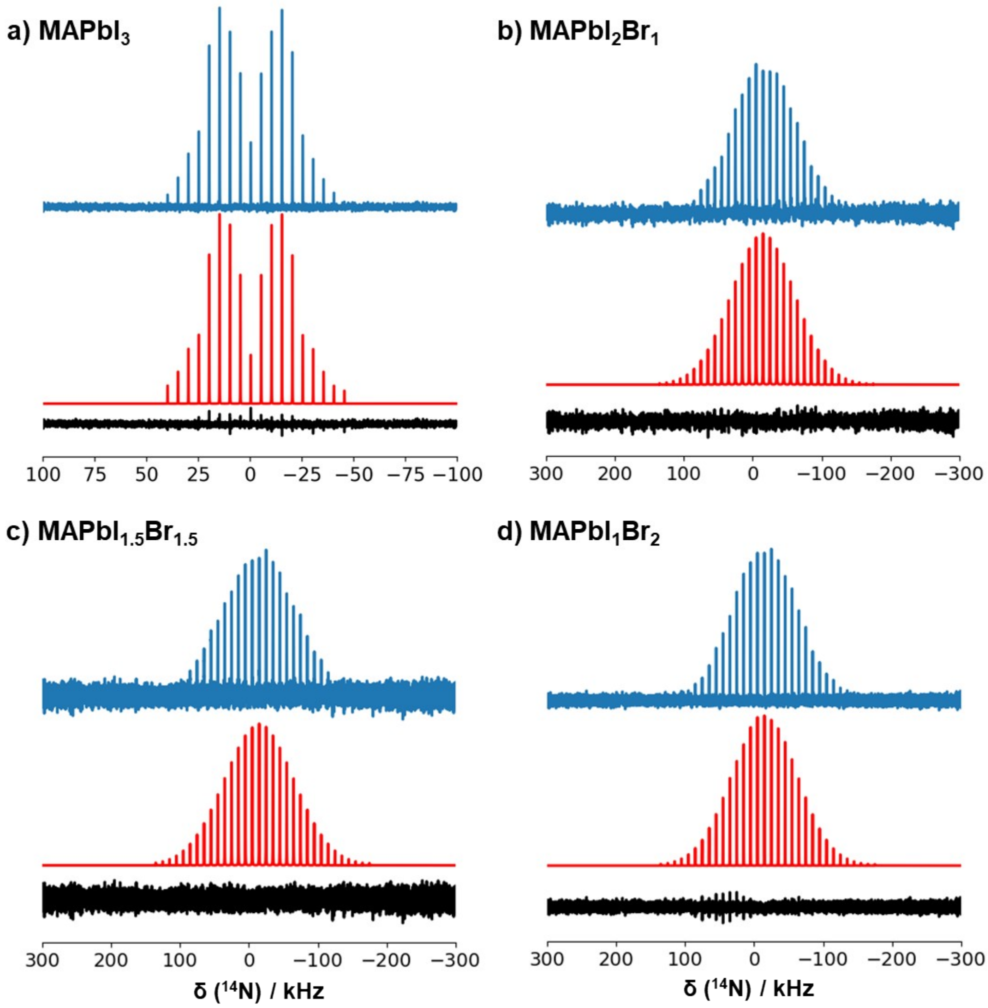

Figure S3. Experimental ^{14}N MAS NMR spectra (blue), corresponding deconvolutions (red) of $MAPbI_3$ (a), $MAPbI_2Br_1$ (b), $MAPbI_{1.5}Br_{1.5}$ (c) and $MAPbI_1Br_2$ (d). The deconvolution was done using a quadrupolar line shape for a) with the values summarized in Tab. S5 and Czjzek[6,7] distributions (compare Fig. S5) for the mixed halide samples in b-d). The difference between experiment and deconvolution is shown in black.

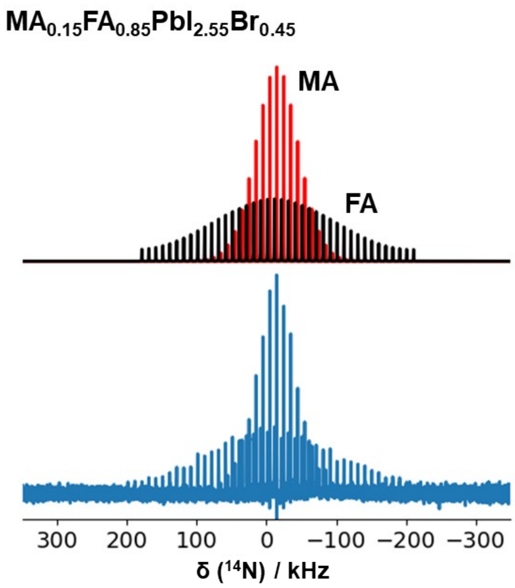

Figure S4. Experimental ^{14}N MAS NMR spectrum (blue) of $MA_{0.15}FA_{0.85}PbI_{2.55}Br_{0.45}$ and corresponding deconvolution using two Czjzek distributions with the values for MA (red) and FA (black) summarized in Tab. S5.

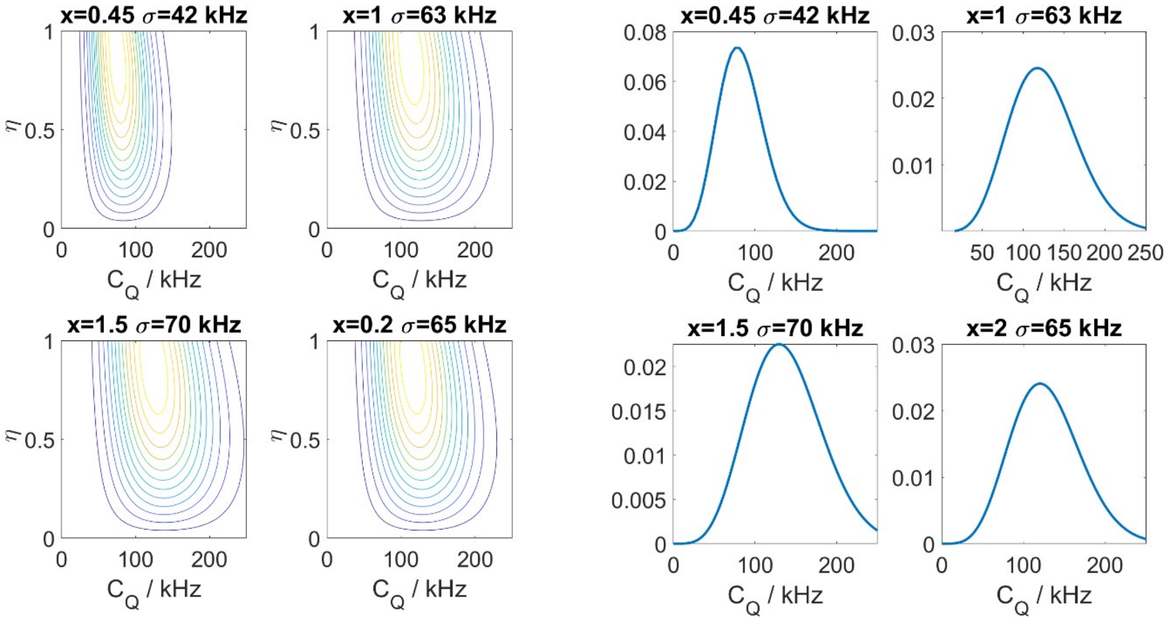

Figure S5. Czjzek distributions of the asymmetry paramter η_Q and quadrupolar coupling C_Q used for the fits in Figs S3 and S4. From these distributions the average quadrupolar coupling $|\bar{C}_Q|$ over the probabilities are extracted and summarized in Tab. S5.

Table S5. Fitting parameters of the ^{14}N quadrupolar line shape depicted in Fig. S3a and the average $|\bar{C}_Q|$ from the Czjzek distribution fits in Fig. S3 and Fig. S4.

	$\|\bar{C}_Q\|$ / kHz	η
**$MAPbI_3$**	55.6± 0.15	0.0
**$MA_{0.15}FA_{0.85}PbI_{2.55}Br_{0.45}$**	MA 83.5 (σ = 42)<br>(FA: 244, σ = 123)	-
**$MAPbI_2Br_1$**	125.2 (σ = 63)	-
**$MAPbI_{1.5}Br_{1.5}$**	136.7 (σ = 70)	-
**$MAPbI_1Br_2$**	127.8 (σ = 65)	-
**$MAPbBr_3$**	0*	-

* from literature ref. [8]

3.3. ^1H DQ NMR spectroscopic data

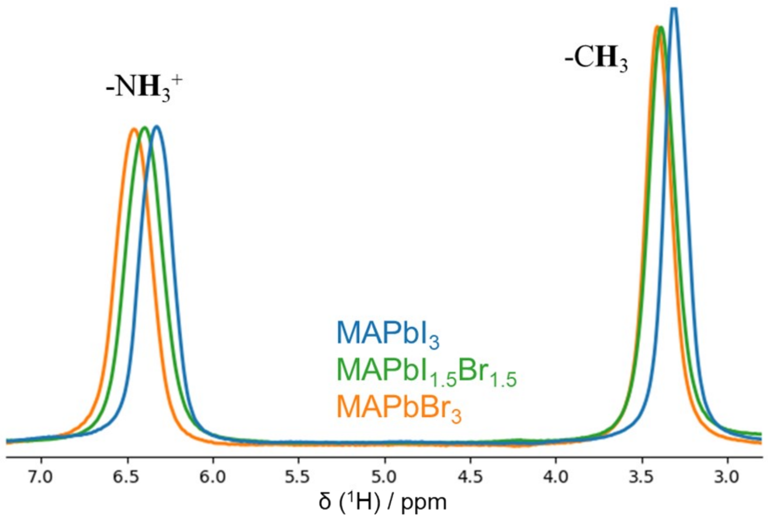

Figure S6. ^1H MAS NMR spectra (recorded at 40 kHz MAS and 850 MHz Lamor frequency) of $MAPbI_3$, $MAPbI_{1.5}Br_{1.5}$ and $MAPbBr_3$. The ^1H chemical shift of the NH_3^+-group shows a slight downfield shift with increasing Br content from 6.32 ppm for pure $MAPbI_3$ to 6.46 ppm for pure $MAPbBr_3$.

Table S6. Experimental average dipolar couplings of the mixed halide and parent perovskites and corresponding lattice constants.

	Experimental $\frac{\bar{D}}{2\pi}$ / Hz	Lattice const. / Å
**$MAPbI_3$**	3232 ± 154	6.269
**$MA_{0.15}FA_{0.85}PbI_{2.55}Br_{0.45}$***	3920 ± 290*	6.30*
**$MAPbI_2Br_1$**	4286 ± 199	6.168
**$MAPbI_{1.5}Br_{1.5}$**	4578 ± 279	6.111
**$MAPbI_1Br_2$**	4256 ± 260	6.041
**$MAPbBr_3$**	3493 ± 111	5.927

* from Ref. [9]

4. First-principles C_Q calculations

The time-averaged EFG is calculated from the MD trajectory for x = 1.5. The EFG is calculated from the self-consistent potential using the method of Petrilli et al.[10] as implemented in VASP. The EFG tensor of each ^{14}N is averaged over the MLFF-MD trajectory and, after this averaging, rotated onto its principal axes to obtain C_Q and η, using the quadrupole moment from the Pyykkö compilation.[11] The EFG tensors are calculated at intervals of 1 ps, in the 4 × 4 × 4 supercell, requiring a total of 355 calculations. In order to keep the computational load manageable we use the PBE functional[12] instead of the SCAN that was used for the ML potential. We used the PBE PAW potentials "Pb", "I", "Br", "C", "N", "H" from the VASP database, so a Pb potential with only 4 unfrozen (valence) electrons. As we do not need to optimize structures and focus on the N electronic structure, this is a reasonable approximation.

Averaging over the 64 ^{14}N in the supercell, we obtain: $\sqrt{\bar{P}_Q^2} = 161\ kHz$, $\bar{C}_Q = 44\ kHz$, $|\bar{C}_Q| = 146\ kHz$, $\bar{\eta} = 0.55$. The effect of time-averaging over the trajectory is considerable: for a purely static, i.e., single time, configuration we observe $\bar{C}_Q = 441\ kHz$, $|\bar{C}_Q| = 763\ kHz$. In Figure S7 the distribution of $|C_Q|$ is plotted, together with a Czjzek[13,14] distribution with the same average $|\bar{C}_Q|$ of 146 kHz. The agreement is very reasonable, given that we only have a sample of 64 nuclei. For an ideal Czjzek distribution one should have $\bar{C}_Q = 0\ kHz$ and $\bar{\eta} = 0.610$. The statistical quality of our simulation data does not allow to discriminate between the Czjzek distribution and possible subtle deviations thereof.

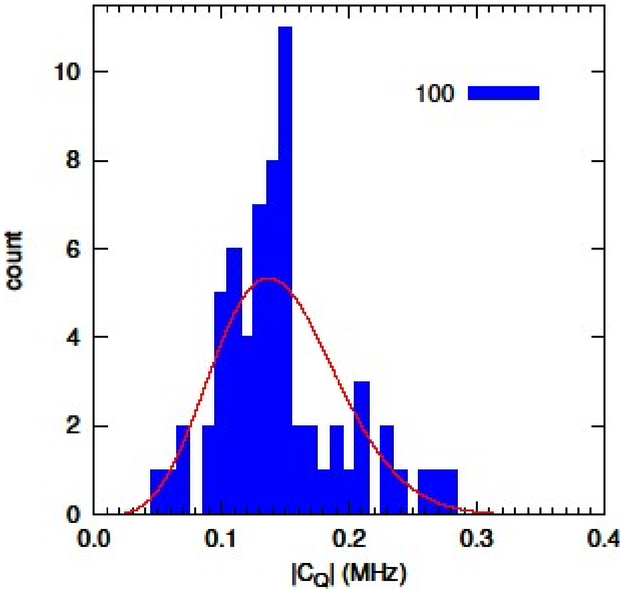

Figure S7. Distribution of C_Q (blue) obtained from time-averaged EFG calculations on the MLFF-MD trajectories compared to a Czjzek distribution (red) with the same $|\bar{C}_Q| = 146\ kHz$.

5. Machine Learning Force Field Method

5.1 MLFF: $MAPbI_{3-x}Br_x$ supercells

The MD simulations have been performed on 4x4x4 supercells containing 64 formula units as shown in Figures S8 and S9. Only the halide ordered systems are shown. The random systems have been generated out of the pure iodine systems, by randomly replacing iodine ions with bromine ions unit the desired fraction x is reached.

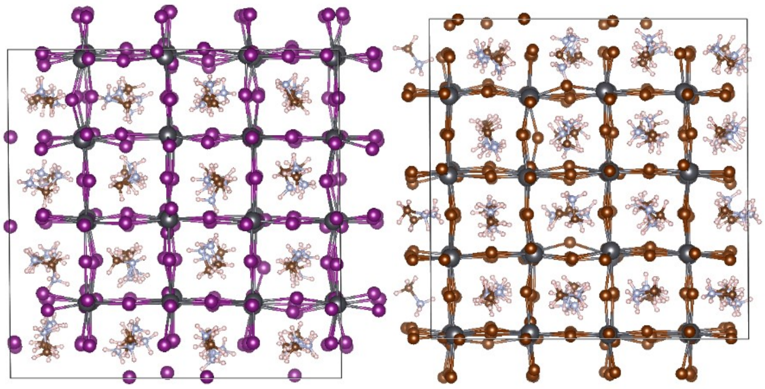

Figure S8. The pure halide x=0 (left) and x=3 (right) supercells.

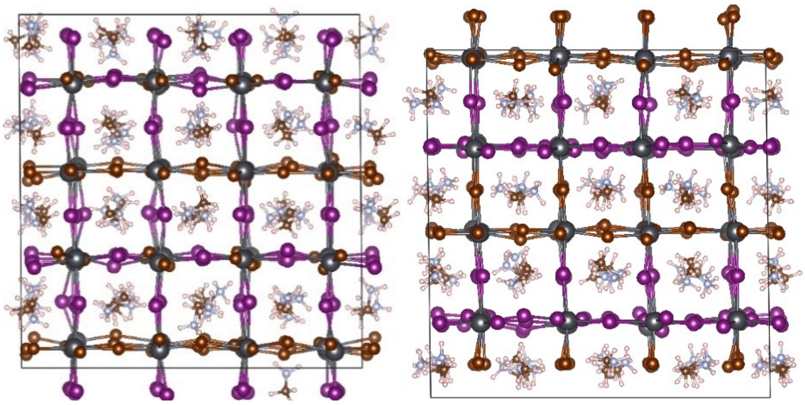

Figure S9. The halide ordered x=1.5 supercells: structure A (left) and B (right).

5.2 MLFF: Calculation of average dipolar coupling and it's relation to second moment

We follow the approach as presented by Goc et. al. to numerically calculate the van Vleck second moment for the dipolar interactions of the protons. Experimentally the average dipolar interaction $\bar{D}$ is extracted from double-quantum built-up curves following the approach by Saalwächter. The van Vleck second moment is defined as [ref. [15] page 112]:

$$M_2 = \frac{3}{4}\left(\frac{\mu_0}{4\pi}\right)^2 \gamma_I^4 \hbar^2 I(I+1) \sum_k \frac{\left(1 - 3\cos^2\theta_{jk}\right)^2}{r_{jk}^6}$$

For a spin I=1/2 and taking the average value of 4/5 for the factor $\overline{\left(1 - 3\cos^2\theta_{jk}\right)^2}$ in a powder, we get:

$$M_2 = \frac{9}{20}\left(\frac{\mu_0}{4\pi}\right)^2 \gamma_I^4 \hbar^2 \sum_k \frac{1}{r_{jk}^6} = \frac{9}{20} D^2$$

As our second moment calculations include both powder averaging and averaging over the dynamics in the MD trajectory, we get $\overline{D}$ from the calculated $\overline{M_2}$:

$$\overline{D} = \sqrt{\frac{20}{9}\overline{M_2}}$$

5.3 MLFF: Fitting D_{intra} and D_{inter}

We have fitted the function $D_{intra} = \left(\alpha(1/t)^{1/n} + \beta\right)$ for each of the seven systems as function of simulation time (t) by a root-mean-square fit. Weight factors have been applied that are linear proportionate to the simulation time length.

x	α / Hz*ps^n	β / Hz	n
A_ordered	$2.45 *10^5$	211.0	3.38
B_segragated	$3.62 *10^5$	7910	1.46
0.000	$3.79 *10^5$	$1.70 *10^{-5}$	2.41
1.125	$2.03 *10^5$	1.183	3.79
1.500	$4.98 *10^5$	3857	1.47
1.875	$7.26 *10^5$	4541	1.09
3.000	$3.19 *10^5$	$1.44 *10^{-2}$	2.08

We have fitted the function $D_{inter} = \left(\alpha(1/t)^{1/n} + \beta\right)$ for each of the seven systems in the same manner as described for D_{intra}.

x	α / Hz*ps	β / Hz	n
A_ordered	$5.89 *10^4$	3628	1.0
B_segragated	$6.05 *10^4$	3763	1.0
0.000	$7.28 *10^4$	3334	1.0
1.125	$4.08 *10^4$	3522	1.0
1.500	$6.69 *10^4$	3558	1.0
1.875	$4.19 *10^4$	3655	1.0
3.000	$5.04 *10^4$	3930	1.0

5.5 Local halide cage description and order parameter

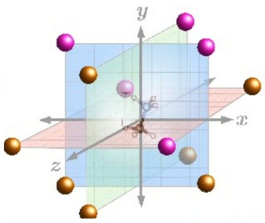

Figure S10. The number of iodines in each of the three planes is used to characterize the local 'cage'.

In an attempt to characterize the halide distributions over these cages with just a few numbers, we introduce an order parameter **O**=[N_{xy},N_{xz},N_{yz}], containing the number of iodine ions (N=0...4) in each of the three cartesian planes through the center of the cage as defined in Figure 4c. This order parameter has been added to each of the 64 environments shown in Figures S13-S17. We further calculate the standard deviation σ^{SB} of its three components, thus expressing the inhomogeneity and asymmetry of the halide coordination of a MA molecule in just a single number. We observe that in general the distributions corresponding to configurations that have a large σ^{SB} (like [124]) show strongly preferred orientations, whereas

configurations with a zero σ^{SB} (like [222]) show a close to flat distribution of the C-N axis orientation, i.e., there is hardly any preferential orientation of the MA cations in such environments. Figs. S16-S18 explore these findings in more detail. They assess the correlation between σ^{SB} and the corrugation of the polar distribution σ^{P}.

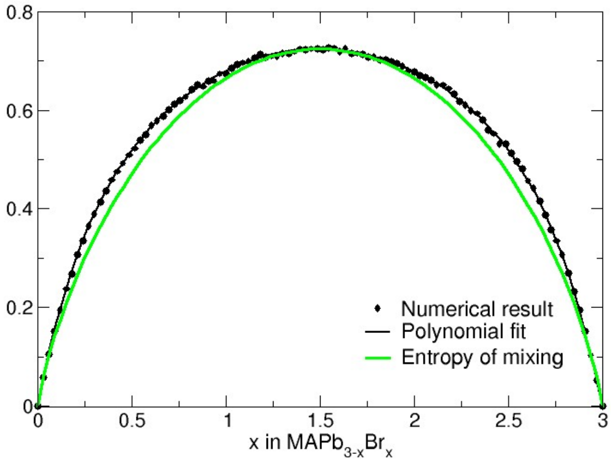

Figure S11. Average variance of the order parameter observed in the infinitely large supercell computed numerically. The entropy of mixing of a binary gas is a good approximate of this function.

We have numerically calculated the function $\sigma^{SB}(x)$ in the limit of very large supercell sizes and plotted the result in Figure S11. Here, we applied the following (Python) routine:

```
numx=100
num=10000
x=np.linspace(0,1,numx)
XYZstd=np.zeros(numx)
for k in range(frac.shape[0]):
  for l in range(num):
    XYZ=np.zeros(3)
    for m in range(3):
      for n in range(4):
        p=rnd.uniform(0,1)
        if p<=x[k]:
          XYZ[m]=XYZ[m]+1
    XYZstd[k]=XYZstd[k]+np.std(XYZ)
XYZstd=XYZstd/num
```

For every x we generate 10,000 random environments [XYZ], calculate the variance of their order parameter and average them. As shown in the figure, the mixing entropy of a binary gas $-m_1(x\ln(x)+(1-x)\ln(1-x))$ multiplied by 1.045 is a good approximate of this numerical result. Note that for the mixing entropy formula the fraction expressed in the range x=[0..1] is used.

5.6 MLFF: Polar distributions of MA molecules

The orientation of each of the 64 molecules in the supercell is extracted as function of MD simulation time. Figure S12 illustrates the $\{\theta,\phi\}$ reference frame in which the orientation is expressed.

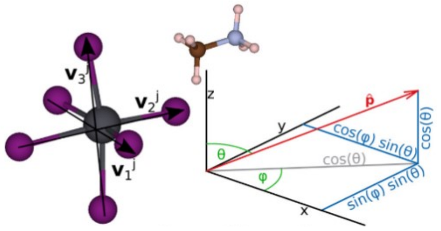

*Figure S12. The cartesian reference frame is aligned along the Pb-X-Pb bonds of the (pseudo)-cubic perovskite framework. The direction of the C-N axis (**p**) is expressed in polar coordinates* $\{\theta,\phi\}$.

The polar distribution plots presented in the main paper as well as in this document are a 2D histogram of the $\{\theta,\phi\}$ as they occur during the MD trajectory. These distributions are normalized and the color scale is set consistent throughout all plots.

Figures S13-S17 show the distribution of each molecules in the:

- Ordered x=1.5 structure A trajectory
- Ordered x=1.5 structure B trajectory
- Random x=1.125 trajectory
- Random x=1.500 trajectory
- Random x=1.875 trajectory

In general, we observe that the MA molecules in the ordered structures show more highly preferred orientations as compared to the randomly distributed halide structures. This means that the molecules in these systems will rotate less isotropically and will therefore result in a higher D_{intra} contribution to the ^1H-^1H dipolar coupling as compared to the random structures.

Note that the 64 distributions are ranked based on the level of anisotropy of the local cage that the particular molecule is in. This level is calculated by the standard deviation of the order parameter. For example, Figure S15 starts with a [333] environment with σ^{SB}=0 and running up to [404] with σ^{SB}=1.89.

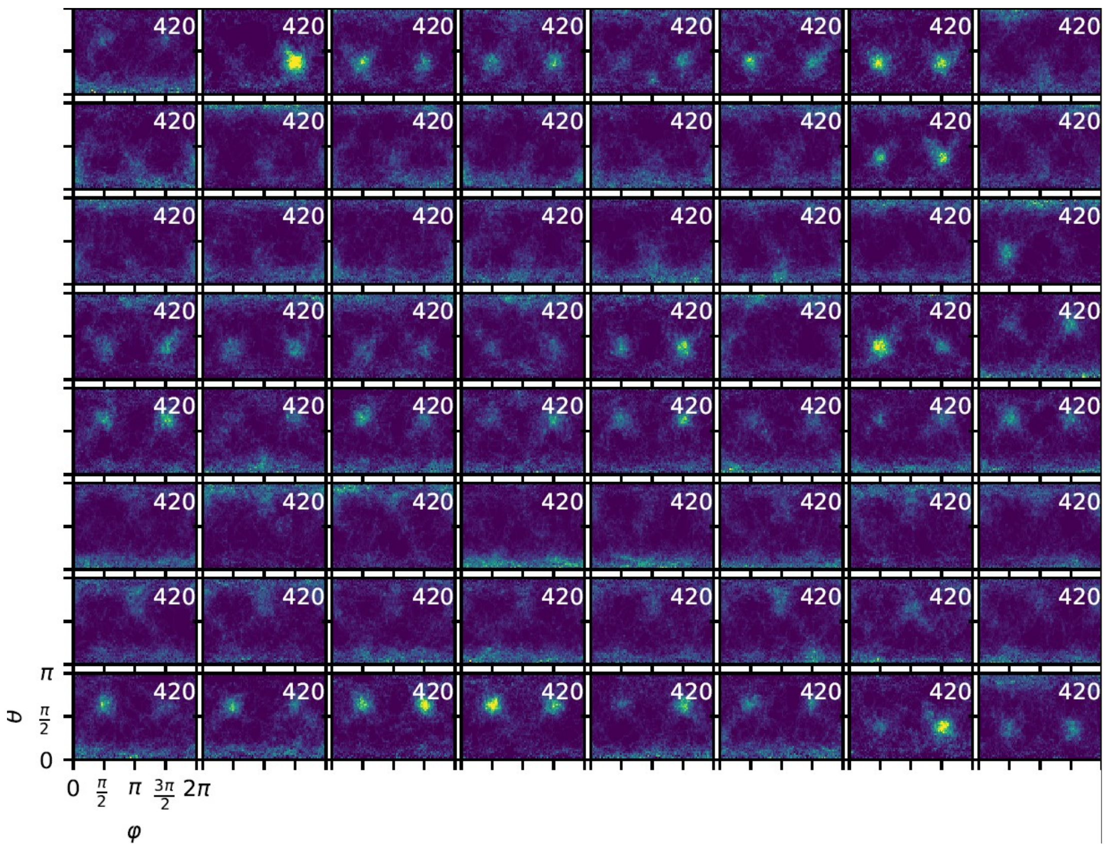

Figure S13. Polar distributions and order parameters of the ordered x=1.5 structure A trajectory.

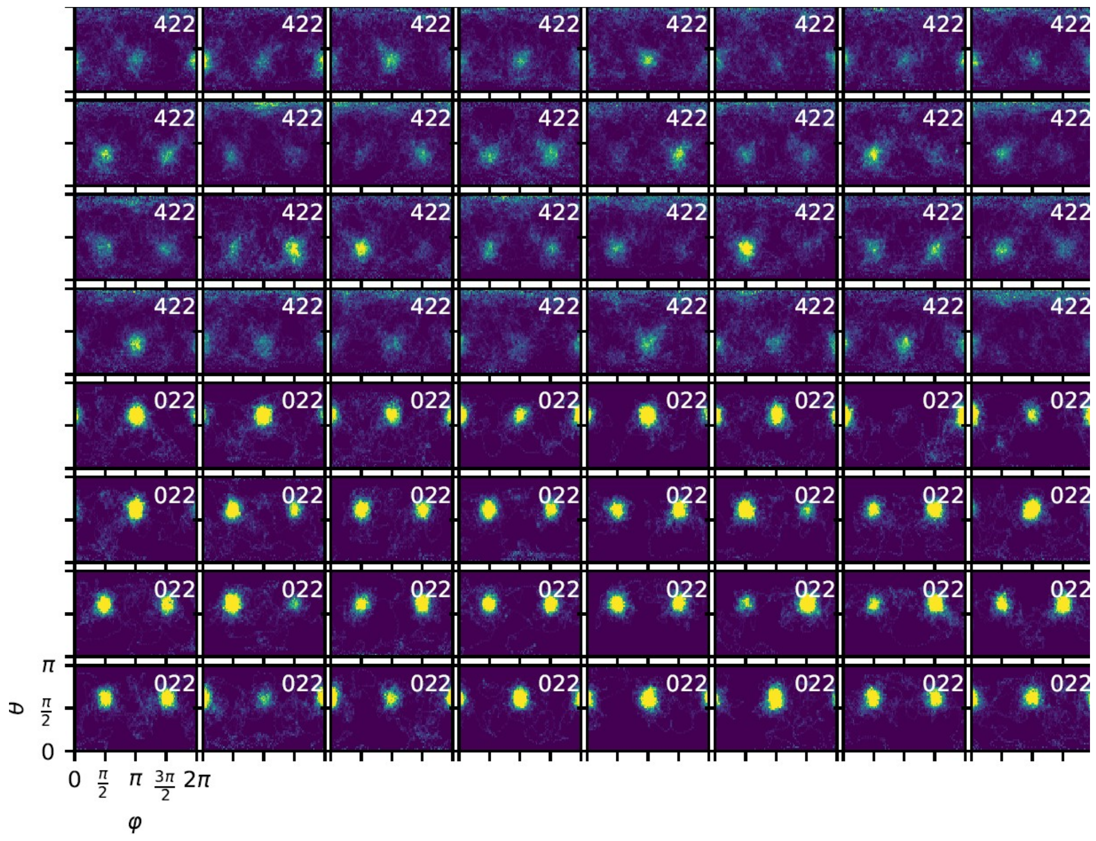

Figure S14. Polar distributions and order parameters of the ordered x=1.5 structure B trajectory.

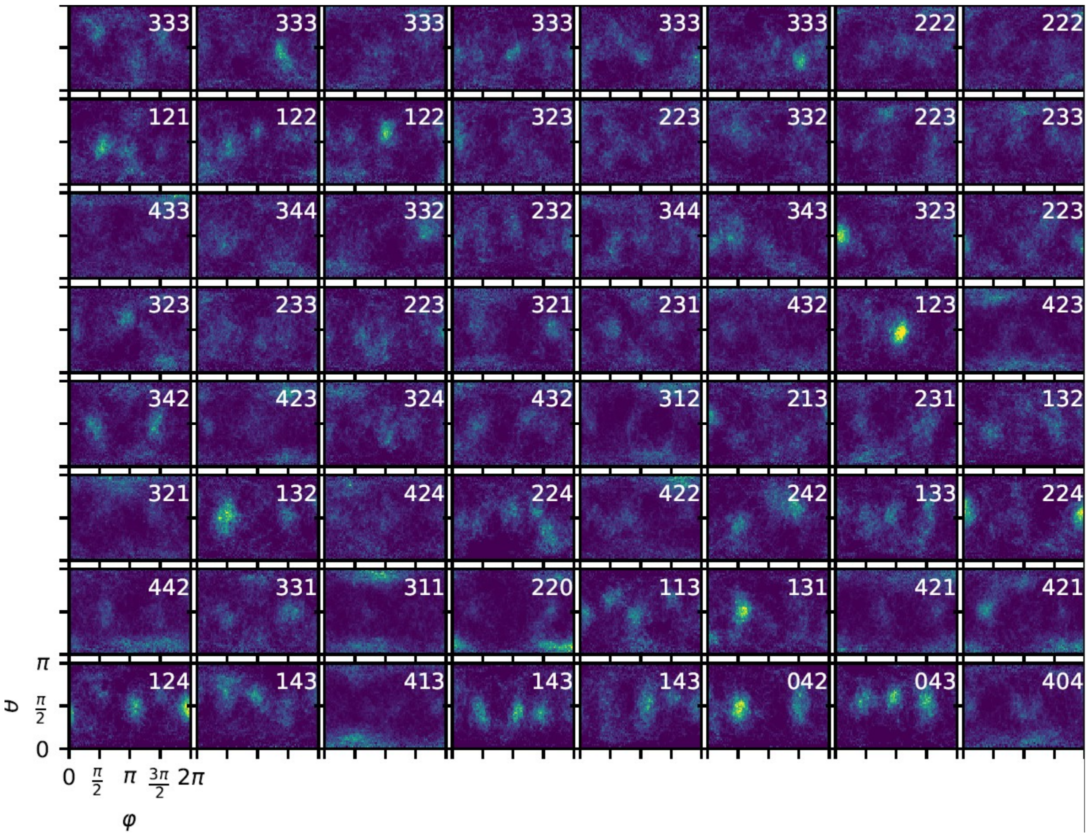

Figure S15. Polar distributions and order parameters of the random x=1.125 structure trajectory.

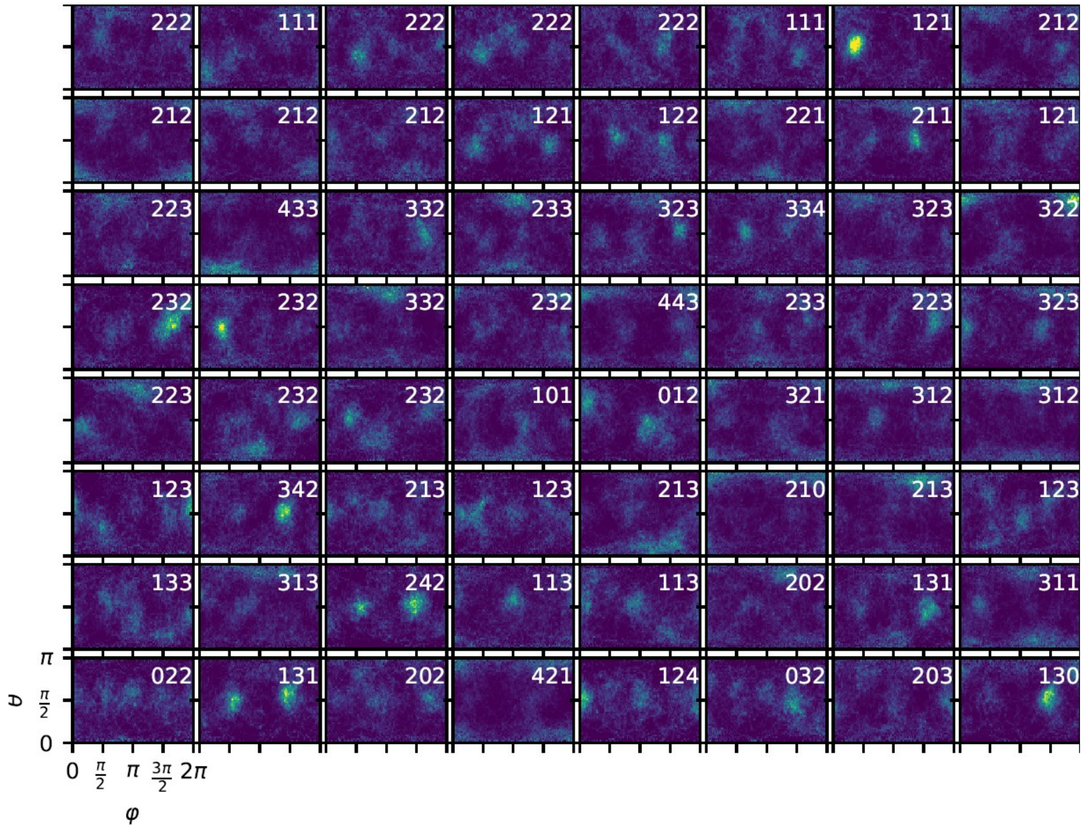

Figure S16. Polar distributions and order parameters of the random x=1.5 structure trajectory.

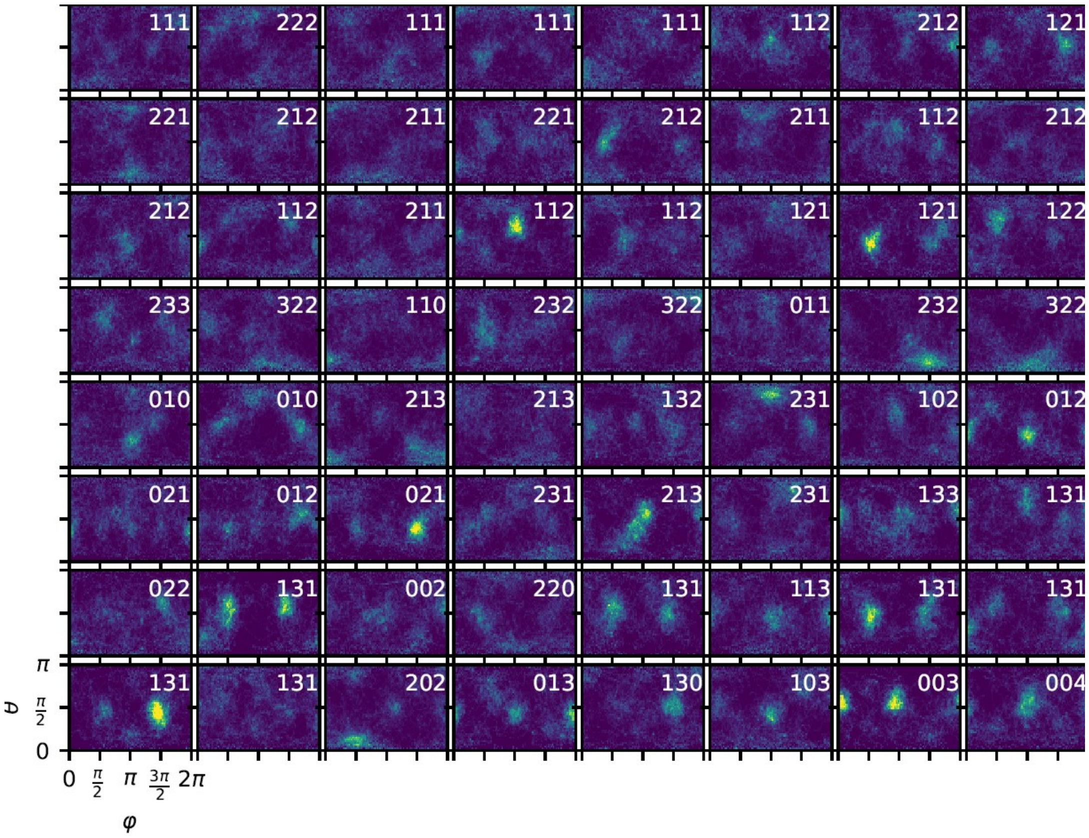

Figure S17. Polar distributions and order parameters of the random x=1.875 structure trajectory.

5.7 MLFF: Dependence of the corrugation of the polar distributions on the order parameter

We will now assume that a large part of the symmetry and corrugation of the polar distribution corresponding to a particular molecule can be related to its local environment of the 12 nearest surrounding halides. We propose the following two physical parameters to model the corrugation level of the polar distribution:

i) Local concentration of the number of Iodines over Bromines [0..1]
ii) Anisotropy level of the local cage [0..3.5]

We have tested the correlation between these two parameters and the variance of the polar distribution. This variance is a direct measure for the corrugation of the polar distribution. As a correlation test the Pearson correlation coefficient is calculated. The resulting coefficients are summarized in Table S7. A value of –1 or 1 would indicate perfect (anti-)correlation. A coefficient of zero indicates no correlation, as in the case of two random sets of numbers. What the coefficients in Table S7 show is that the local concentration does not correlate with the level of corrugation. For the three different supercells different signs of the Pearson coefficients are observed. However, the level of anisotropy does correlate with the level of corrugation, and shows positive Pearson coefficients that increase with the global Bromide fraction. Note that these correlation coefficients are small, but statistically significant. It illustrates that the composition of the local cage is important, but the level of anisotropy is not fully sufficient to predict the polar distribution of a molecule.

Table S7. Pearson correlation coefficients describing the level of correlation between the corrugation of the polar distribution of individual molecules with the i) concentration of Iodine x and ii) the level of anisotropy in the local surrounding halide cage.

x in $MAPbI_{3-x}Br_x$	Local concentration x	Level of Anisotropy
1.125	-0.110	0.073
1.500	0.065	0.274
1.875	-0.147	0.387

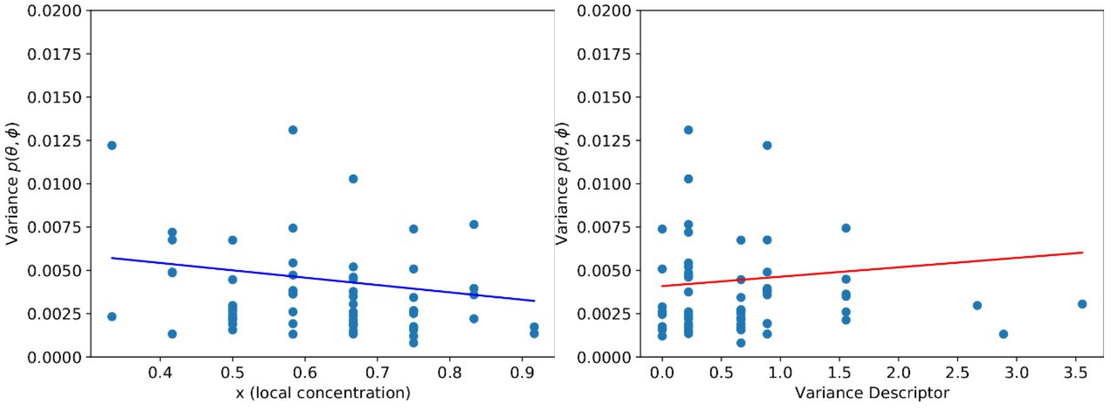

Figure S18. Variance of the polar distributions versus i) local concentration (blue) and ii) variance of the order parameters (σ^{SB}) (red) of the random x=1.125 structure trajectory.

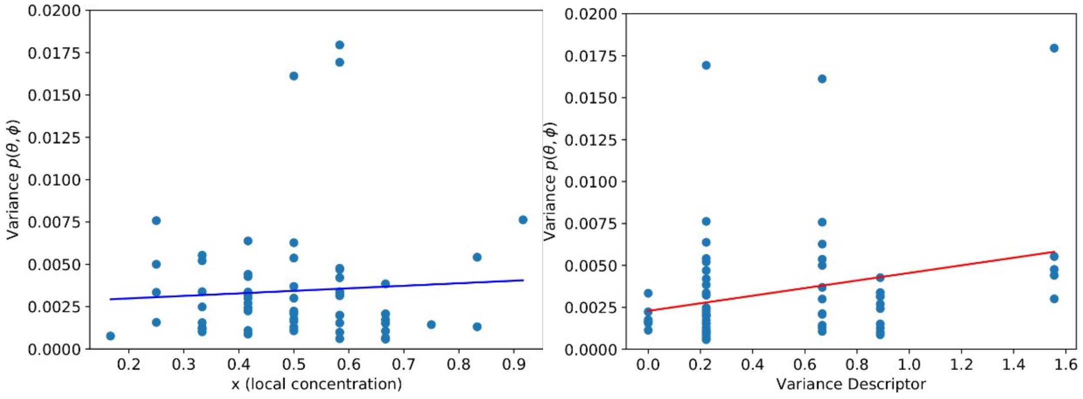

Figure S19. Variance of the polar distributions versus i) local concentration (blue) and ii) variance of the order parameters (σ^{SB}) (red) of the random x=1.5 structure trajectory.

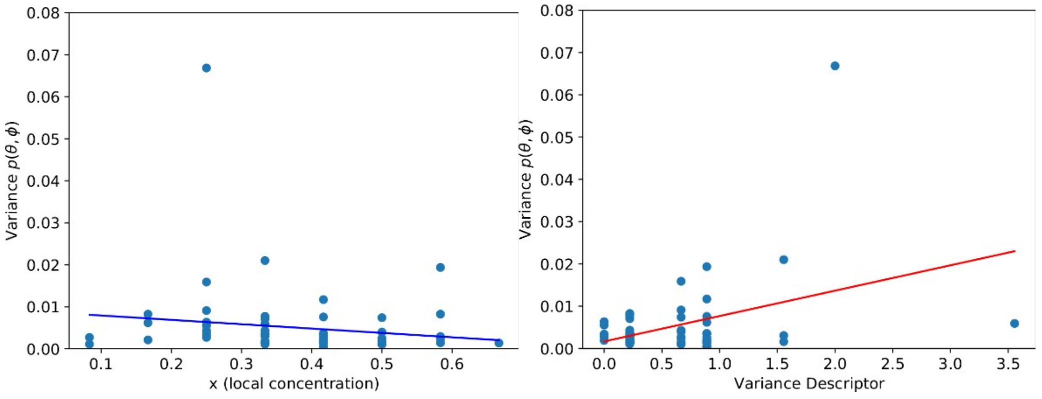

Figure S20. Variance of the polar distributions versus i) local concentration (blue) and ii) variance of the order parameters (σ^{SB}) (red) of the random x=1.875 structure trajectory.

The least-mean-square fitted red linear curve in Figures S18-S20 clearly shows that there is a correlation between the corrugation, and therefore the level of non-isotropy, of the polar distribution of the molecule depending on the local environment that it is in. Therefore, it is a legitimate descriptor to model the level of non-isotropy of the polar distribution. As expected, we also see that this simple descriptor (the local order parameter **O**) is not perfect. There is a large spread in the level of corrugation of polar distributions assigned to environments that have the same σ^{SB} value. Note that **O** does not describe how the I and Br ions are distributed in the cartesian planes, nor does not take into account the type of the nearest neighbour environments. Of course, all these effects could be included, but it will greatly increase the complexity of the order parameter.

5.8 MLFF: Reorientation dynamics of MA molecules

The autocorrelation functions of the molecular orientations **p**(t) as sketched in Figure S12 are calculated for all molecules in the random structures. This is done in the same manner as explained in Ref. [16]. The autocorrelation functions (blue lines) are shown in Figures S22-S24 and fitted with a double exponential (orange lines). The fit is very good for all molecules in the x=1.125 and 1.5 random systems, and sufficient in the x=1.875 system. Distributions of all fit parameters are shown in Figure S21. In all cases we observe a fast 'thermal' decorrelation process of between 0.5-3 ps and a slow 'flipping' decorrelation process of between 3-30 ps.

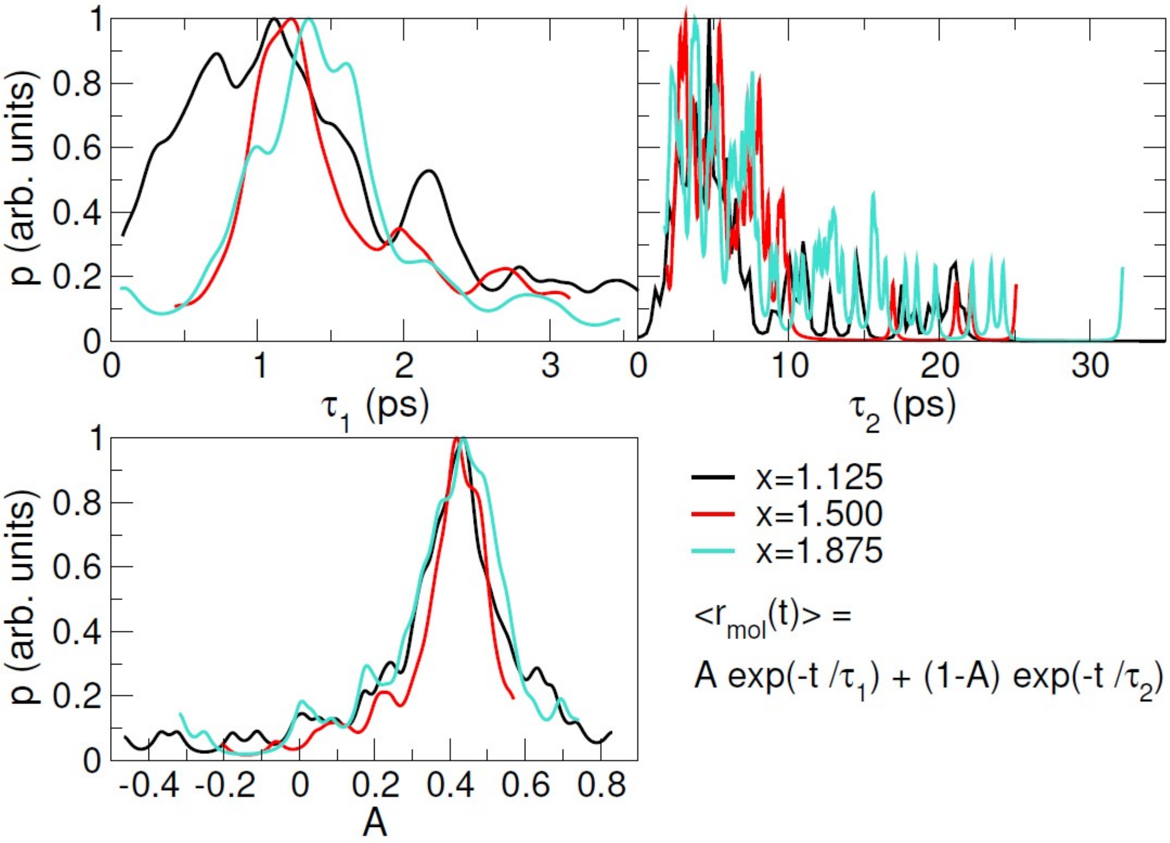

Figure S21. Distributions of parameters obtained from fitting each of the 64 autocorrelation functions corresponding to the molecules in in the x=1.125, 1.500 and 1.875 randomly mixed halides structures.

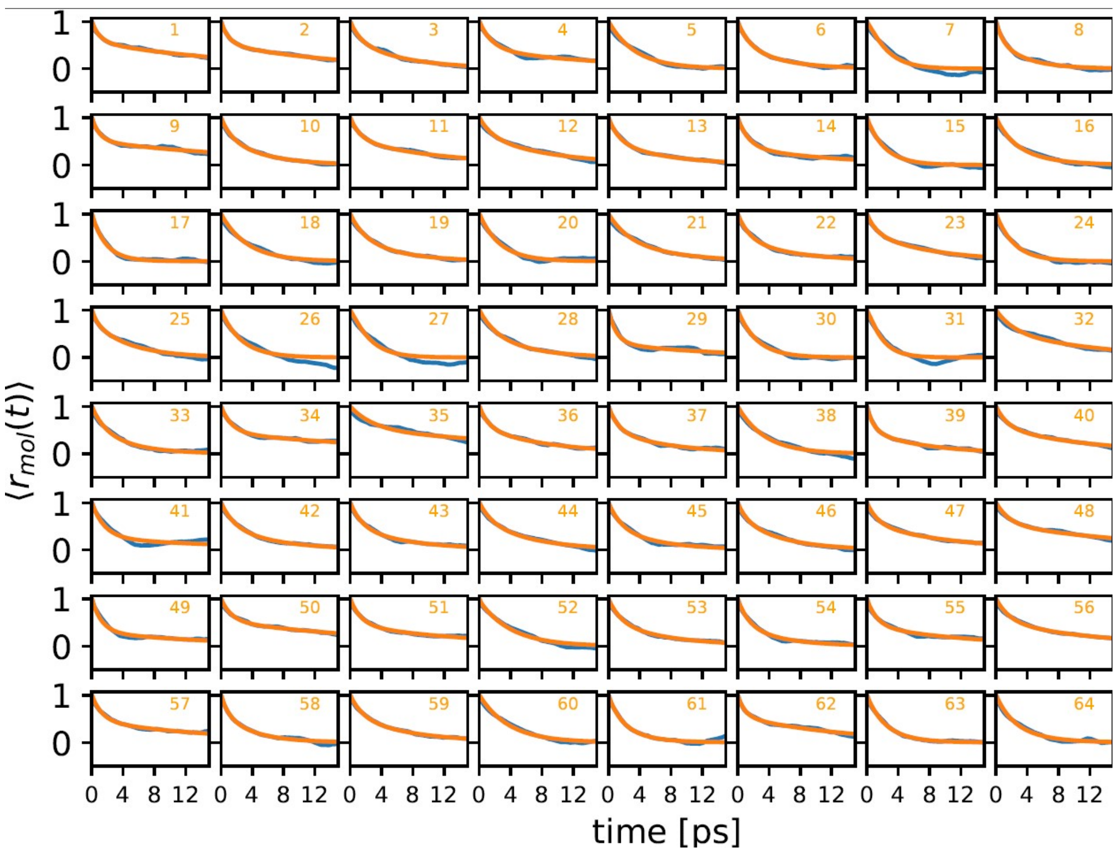

Figure S22. Autocorrelation functions (blue) and their fits (orange) of each of the 64 molecules in the x=1.125 randomly mixed halides structure.

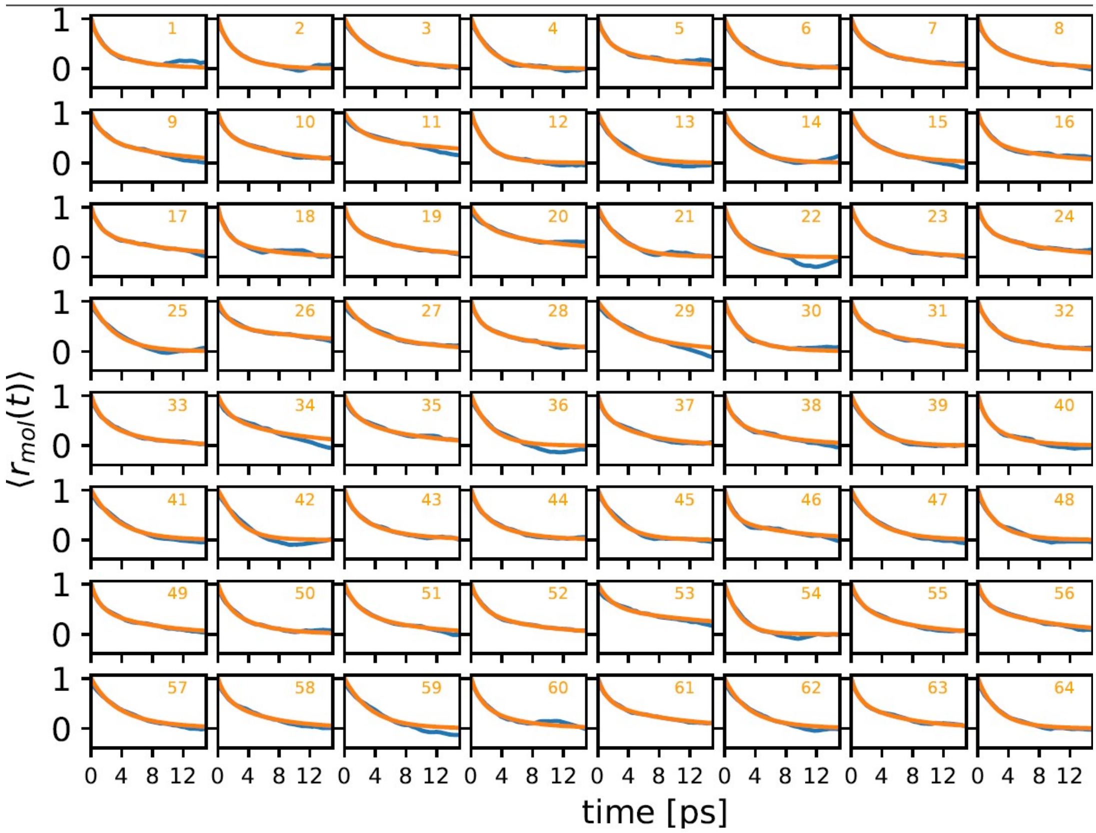

Figure S23. Autocorrelation functions (blue) and their fits (orange) of each of the 64 molecules in the x=1.5 randomly mixed halides structure.

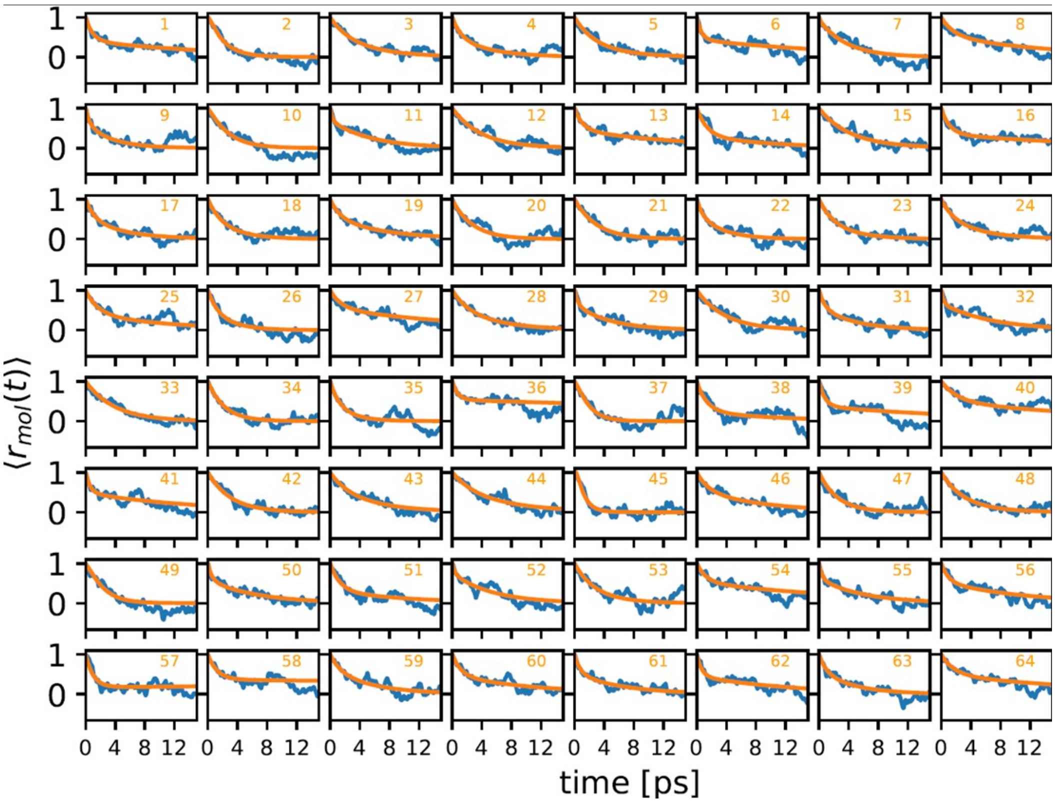

Figure S24. Autocorrelation functions (blue) and their fits (orange) of each of the 64 molecules in the x=1.875 randomly mixed halides structure.

6. SI References

1 A. Karmakar, A. M. Askar, G. M. Bernard, V. V. Terskikh, M. Ha, S. Patel, K. Shankar and V. K. Michaelis, *Chem. Mater.*, 2018, **30**, 2309–2321.

2 A. M. Askar, A. Karmakar, G. M. Bernard, M. Ha, V. V. Terskikh, B. D. Wiltshire, S. Patel, J. Fleet, K. Shankar and V. K. Michaelis, *J. Phys. Chem. Lett.*, 2018, **9**, 2671–2677.

3 A. Karmakar, M. S. Dodd, X. Zhang, M. S. Oakley, M. Klobukowski and V. K. Michaelis, *Chem. Commun.*, 2019, **55**, 5079–5082.

4 W. M. J. Franssen, S. G. D. Van Es, R. Dervişoğlu, G. A. de Wijs and A. P. M. Kentgens, *J. Phys. Chem. Lett.*, 2017, **8**, 61–66.

5 C. Roiland, G. Trippé-Allard, K. Jemli, B. Alonso, J.-C. C. Ameline, R. Gautier, T. Bataille, L. Le Pollès, E. Deleporte, J. Even and C. Katan, *Phys. Chem. Chem. Phys.*, 2016, **18**, 27133–27142.

6 P. J. Knijn, P. J. M. van Bentum, E. R. H. van Eck, C. Fang, D. L. A. G. Grimminck, R. A. de Groot, R. W. A. Havenith, M. Marsman, W. L. Meerts, G. A. De Wijs and A. P. M. Kentgens, *Phys. Chem. Chem. Phys.*, 2010, **12**, 11517–11535.

7 J.-B. d'Espinose de Lacaillerie, C. Fretigny and D. Massiot, *J. Magn. Reson.*, 2008, **192**, 244–251.

8 O. Knop, R. E. Wasylishen, M. A. White, T. S. Cameron and M. J. M. Van Oort, *Can. J. Chem.*, 1990, **68**, 412–422.

9 H. Grüninger, M. Bokdam, N. Leupold, P. Tinnemans, R. Moos, G. A. De Wijs, F. Panzer and A. P. M. M. Kentgens, *J. Phys. Chem. C*, 2021, **125**, 1742–1753.

10 H. M. Petrilli, P. E. Blöchl, P. Blaha and K. Schwarz, *Phys. Rev. B*, 1998, **57**, 14690–14697.

11 P. Pyykkö, *Mol. Phys.*, 2018, **116**, 1328.

12 J. P. Perdew, K. Burke and M. Ernzerhof, *Phys. Rev. Lett.*, 1996, **77**, 3865–3868.

13 G. Czjzek, J. Fink, F. Götz, H. Schmidt, J. M. D. Coey, J.-P. Rebouillat and A. Liénard, *Phys. Rev. B*, 1981, **23**, 2513–2530.

14 U. Werner-Zwanziger, A. L. Paterson and J. W. Zwanziger, *J. Non. Cryst. Solids*, 2020, **550**, 120383.

15 A. Abragam, *The Principles of Nuclear Magnetism*, Oxford, 1961.

16 J. Lahnsteiner, G. Kresse, J. Heinen and M. Bokdam, *Phys. Rev. Mater.*, 2018, **2**, 073604.
